# Supplementary material for: Geographical Origin Does Not Modulate Pathogenicity or Response to Climatic Variables of Fusarium oxysporum Associated with Vascular Wilt on Asparagus
Source: J Fungi (Basel). 2021 Dec 9;7(12):1056. doi: 10.3390/jof7121056 (PMC8703408; doi:10.3390/jof7121056)
Supplement: Supplementary file 1 [file jof-07-01056-s001.zip › jof-1443396-supplementary.pdf]

S1: Final alignments in FASTA format (TEF1A)

```
>fusarium_proliferatum_et1_gca_900067095
gcgattcgaaacgtgcctgctaccccgctcgagaccaaaatttttgcgatatgaccgtaa
-tttttttggtggggcatttaccccgccactcgagcgatgagcgcgtttttgcccctt--t
cctgtccacaacctcaatgagcgcattgtcacgtgtcaagcagcgactaaccattcgaca
ataggaagccgctgagctcggtaagggttccttcaagtacgcctgggttcttgacaagct
caaggccgagcgtgagcgtggtatcaccatcgatattgctctctggaagttcgagactcc
tcgctactatgtcaccgtcattggtatgttgtcgctcatacctcatcctact--tcctca
tactaacacatcattcagacgctcccggtcaccgtgatttcatcaaga
>fusarium_oxysporum_fo47_gca_000271705
acgactcgaaacgtgcccgctaccccgctcgagaccaaaaattttgcaatatgactgtaa
ttttttttggtggggcacttaccccgccacttgagcgacgggagcgtttgccctcttaac
cattctcacaacctcaatgagtgcgtcgtcacgtgtcaagcagtcactaaccattcaaca
ataggaagccgctgagctcggtaagggttccttcaagtacgcctgggttcttgacaagct
caaggccgagcgtgagcgtggtatcaccatcgatattgctctctggaagttcgagactcc
tcgctactatgtcaccgtcattggtatgttgtcgctcatgcttcattctacttctcttcg
tacta--acatcactcagacgctcccggtcaccgtgatttcatcaaga
>GASP_2
acgactcgaaacgtgcccgctaccccgctcgagaccaagaatcttgcaatatgaccgtaa
-tttttttggtggggcacttaccccgccacttgagcgacgggagcgtttgccctcttaac
cattctcacaacctcaatgagtgcgtcgtcacgtgtcaagcagtcactaaccattcaaca
ataggaagccgctgagctcggtaagggttccttcaagtacgcctgggttcttgacaagct
caaggccgagcgtgagcgtggtatcaccatcgatattgctctctggaagttcgagactcc
tcgctactatgtcaccgtcattggtatgttgtcgctcatgcttcattctacttctcttcg
tacta--acatcactcagacgctcccggtcaccgtgatttcatcaaga
>GASP_3
acgactcgaaacgtgcccgctaccccgctcgagaccaaaaattttgcaatatgactgtaa
ttttttttggtggggcacttaccccgccacttgagcgacgggagcgtttgccctcttaac
cattctcacaacctcaatgagtgcgtcgtcacgtgtcaagcagtcactaaccattcaaca
ataggaagccgctgagctcggtaagggttccttcaagtacgcctgggttcttgacaagct
caaggccgagcgtgagcgtggtatcaccatcgatattgctctctggaagttcgagactcc
tcgctactatgtcaccgtcattggtatgttgtcgctcatgcttcattctacttctcttcg
tacta--acatcactcagacgctcccggtcaccgtgatttcatcaaga
>GASP_4
acgactcgaaacgtgcccgctaccccgctcgagaccaaaaattttgcaatatgaccgtaa
tttttttcggtggggcacttaccccgccacttgagcgacgggagcgtttgccctctt-ac
cattctcacaacctcaatgagtgcgtcgtcacgtgtcaagcagtcactaaccattcaaca
ataggaagccgctgagctcggtaagggttccttcaagtacgcctgggttcttgacaagct
caaggccgagcgtgagcgtggtatcaccatcgatattgctctctggaagttcgagactcc
tcgctactatgtcaccgtcattggtatgttgtcgttcatgcttcattctacttctcttcg
tacta--acatcactcagacgctcccggtcaccgtgatttcatcaaga
>GASP_5
---------aacgtgcccgctaccccgctcgagaccaaaaattttgcaatatgaccgtaa
-tttttttggtggggcacttaccccgccacttgagcgacgggagcgtttgccctcttaac
cattctcataacctcaatgagtgcgtcgtcacgtgtcaagcagtcactaaccattcaaca
ataggaagccgctgagctcggtaagggttccttcaagtacgcctgggttcttgacaagct
caaggccgagcgtgagcgtggtatcaccatcgatattgctctctggaagttcgagactcc
tcgctactatgtcaccgtcattggtatgttgtcgctcaagcttcattctacttctcttcg
tacta--acatcactcagacgctcccggtcaccgtgatttcatcaaga
>GASP_9
acgactcgaaacgtgcccgctaccccgctcgagaccaaaaattttgcaatatgactgtaa
ttttttttggtggggcacttaccccgccacttgagcgacgggagcgtttgccctcttaac
cattctcacaacctcaatgagtgcgtcgtcacgtgtcaagcagtcactaaccattcaaca
ataggaagccgctgagctcggtaagggttccttcaagtacgcctgggttcttgacaagct
caaggccgagcgtgagcgtggtatcaccatcgatattgctctctggaagttcgagactcc
tcgctactatgtcaccgtcattggtatgttgtcgctcatgcttcattctacttctcttcg
tacta--acatcactcagacgctcccggtcaccgtgatttcatcaaga
>GA_1.2
acgactcgaaacgtgcccgctaccccgctcgagaccaagaatcttgcaatatgaccgtaa
-tttttttggtggggcacttaccccgccacttgagcgacgggagcgtttgccctcttaac
```

```
cattctcacaacctcaatgagtgcgtcgtcacgtgtcaagcagtcactaaccattcaaca
ataggaagccgctgagctcggtaagggttccttcaagtacgcctgggttcttgacaagct
caaggccgagcgtgagcgtggtatcaccatcgatattgctctctggaagttcgagactcc
tcgctactatgtcaccgtcattggtatgttgtcgctcatgcttcattctacttctcttcg
tacta--acatcactcagacgctcccggtcaccgtgatttcatcaaga
>GA_1.3
acgactcgaaacgtgcccgctaccccgctcgagaccaaaaattttgcaatatgactgtaa
ttttttttggtggggcacttaccccgccacttgagcgacgggagcgtttgccctcttaac
cattctcacaacctcaatgagtgcgtcgtcacgtgtcaagcagtcactaaccattcaaca
ataggaagccgctgagctcggtaagggttccttcaagtacgcctgggttcttgacaagct
caaggccgagcgtgagcgtggtatcaccatcgatattgctctctggaagttcgagactcc
tcgctactatgtcaccgtcattggtatgttgtcgctcatgcttcattctacttctcttcg
tacta--acatcactcagacgctcccggtcaccgtgatttcatcaaga
>GA_1.4
acgactcgaaacgtgcccgctaccccgctcgagaccaagaatcttgcaatatgaccgtaa
-tttttttggtggggcacttaccccgccacttgagcgacgggagcgtttgccctcttaac
cattctcacaacctcaatgagtgcgtcgtcacgtgtcaagcagtcactaaccattcaaca
ataggaagccgctgagctcggtaagggttccttcaagtacgcctgggttcttgacaagct
caaggccgagcgtgagcgtggtatcaccatcgatattgctctctggaagttcgagactcc
tcgctactatgtcaccgtcattggtatgttgtcgctcatgcttcattctacttctcttcg
tacta--acatcactcagacgctcccggtcaccgtgatttcatcaaga
>GA_10
acgactcgaagcgtgcccgctaccccgctcgagaccaagaatcttgcaatatgaccgtaa
-tttttttggtggggcacttaccccgccacttgagcgacgggagcgtttgccctcttaac
cattctcacaacctcaatgagtgcgtcgtcacgtgtcaagcagtcactaaccattcaaca
ataggaagccgctgagctcggtaagggttccttcaagtacgcctgggttcttgacaagct
caaggccgagcgtgagcgtggtatcaccatcgatattgctctctggaagttcgagactcc
tcgctactatgtcaccgtcattggtatgttgtcgctcatgcttcattctacttctcttcg
tacta--acatcactcagacgctcccggtcaccgtgatttcatcaaga
>GA_3.2
acgactcgaaacgtgcccgctaccccgctcgagaccaaaaattttgcaatatgactgtaa
ttttttttggtggggcacttaccccgccacttgagcgacgggagcgtttgccctcttaac
cattctcacaacctcaatgagtgcgtcgtcacgtgtcaagcagtcactaaccattcaaca
ataggaagccgctgagctcggtaagggttccttcaagtacgcctgggttcttgacaagct
caaggccgagcgtgagcgtggtatcaccatcgatattgctctctggaagttcgagactcc
tcgctactatgtcaccgtcattggtatgttgtcgctcatgcttcattctacttctcttcg
tacta--acatcactcagacgctcccggtcaccgtgatttcatcaaga
>GA_3.3
acgactcgaagcgtgcccgctaccccgctcgagaccaagaatcttgcaatatgaccgtaa
-tttttttggtggggcacttaccccgccacttgagcgacgggagcgtttgccctcttaac
cattctcacaacctcaatgagtgcgtcgtcacgtgtcaagcagtcactaaccattcaaca
ataggaagccgctgagctcggtaagggttccttcaagtacgcctgggttcttgacaagct
caaggccgagcgtgagcgtggtatcaccatcgatattgctctctggaagttcgagactcc
tcgctactatgtcaccgtcattggtatgttgtcgctcatgcttcattctacttctcttcg
tacta--acatcactcagacgctcccggtcaccgtgatttcatcaaga
>GA_3.5
acgactcgaaacgtgcccgctaccccgctcgagaccaagaatcttgcaatatgaccgtaa
-tttttttggtggggcacttaccccgccacttgagcgacgggagcgtttgccctcttaac
cattctcacaacctcaatgagtgcgtcgtcacgtgtcaagcagtcactaaccattcaaca
ataggaagccgctgagctcggtaagggttccttcaagtacgcctgggttcttgacaagct
caaggccgagcgtgagcgtggtatcaccatcgatattgctctctggaagttcgagactcc
tcgctactatgtcaccgtcattggtatgttgtcgctcatgcttcattctacttctcttcg
tacta--acatcactcagacgctcccggtcaccgtgatttcatcaaga
>GA_3.6
acgactcgaaacgtgcccgctaccccgctcgagaccaaaaattttgcaatatgaccgtaa
-tttttttggtggggcacttaccccgccacttgagcgacgggagcgtttgccctcttaac
cattctcacaacctcaatgagtgcgtcgtcacgtgtcaagcagtcactaaccattcaaca
ataggaagccgctgagctcggtaagggttccttcaagtacgcctgggttcttgacaagct
caaggccgagcgtgagcgtggtatcaccatcgatattgctctctggaagttcgagactcc
tcgctactatgtcaccgtcattggtatgttgtcgctcatgcttcattctacttctcttcg
tacta--acatcactcagacgctcccggtcaccgtgatttcatca---
```

>GA_3.7
acgactcgaagcgtgcccgctaccccgctcgagaccaagaatcttgcaatatgaccgtaa
-tttttttggtggggcacttaccccgccacttgagcgacgggagcgtttgccctcttaac
cattctcacaacctcaatgagtgcgtcgtcacgtgtcaagcagtcactaaccattcaaca
ataggaagccgctgagctcggtaagggttccttcaagtacgcctgggttcttgacaagct
caaggccgagcgtgagcgtggtatcaccatcgatattgctctctggaagttcgagactcc
tcgctactatgtcaccgtcattggtatgttgtcgctcatgcttcattctacttctcttcg
tacta--acatcactcagacgctcccggtcaccgtgatttcatcaaga
>GA_4.1
acgactcgaagcgtgcccgctaccccgctcgagaccaagaatcttgcaatatgaccgtaa
-tttttttggtggggcacttaccccgccacttgagcgacgggagcgtttgccctcttaac
cattctcacaacctcaatgagtgcgtcgtcacgtgtcaagcagtcactaaccattcaaca
ataggaagccgctgagctcggtaagggttccttcaagtacgcctgggttcttgacaagct
caaggccgagcgtgagcgtggtatcaccatcgatattgctctctggaagttcgagactcc
tcgctactatgtcaccgtcattggtatgttgtcgctcatgcttcattctact--tcttcg
tactaacatatcactcagacgctcccggtcaccgtgatttcatcaaga
>GA_4.2
acgactcgaaacgtgcccgctaccccgctcgagaccaaaaattttgcaatatgactgtaa
ttttttttggtggggcacttaccccgccacttgagcgacgggagcgtttgccctcttaac
cattctcacaacctcaatgagtgcgtcgtcacgtgtcaagcagtcactaaccattcaaca
ataggaagccgctgagctcggtaagggttccttcaagtacgcctgggttcttgacaagct
caaggccgagcgtgagcgtggtatcaccatcgatattgctctctggaagttcgagactcc
tcgctactatgtcaccgtcattggtatgttgtcgctcatgcttcattctacttctcttcg
tacta--acatcactcagacgctcccggtcaccgtgatttcatcaaga
>GA_4.3
acgactcgaagcgtgcccgctaccccgctcgagaccaagaatcttgcaatatgaccgtaa
-tttttttggtggggcacttaccccgccacttgagcgacgggagcgtttgccctcttaac
cattctcacaacctcaatgagtgcgtcgtcacgtgtcaagcagtcactaaccattcaaca
ataggaagccgctgagctcggtaagggttccttcaagtacgcctgggttcttgacaagct
caaggccgagcgtgagcgtggtatcaccatcgatattgctctctggaagttcgagactcc
tcgctactatgtcaccgtcattggtatgttgtcgctcatgcttcattctact--tcttcg
tactaacatatcactcagacgctcccggtcaccgtgatttcatcaaga
>GA_5.3
acgactcgaaacgtgcccgctaccccgctcgagaccaaaaattttgcaatatgactgtaa
ttttttttggtggggcacttaccccgccacttgagcgacgggagcgtttgccctcttaac
cattctcacaacctcaatgagtgcgtcgtcacgtgtcaagcagtcactaaccattcaaca
ataggaagccgctgagctcggtaagggttccttcaagtacgcctgggttcttgacaagct
caaggccgagcgtgagcgtggtatcaccatcgatattgctctctggaagttcgagactcc
tcgctactatgtcaccgtcattggtatgttgtcgctcatgcttcattctacttctcttcg
tacta--acatcactcagacgctcccggtcaccgtgatttcatcaaga
>GA_5.4
acgactcgaaacgtgcccgctaccccgctcgagaccaaaaattttgcaatatgactgtaa
ttttttttggtggggcacttaccccgccacttgagcgacgggagcgtttgccctcttaac
cattctcacaacctcaatgagtgcgtcgtcacgtgtcaagcagtcactaaccattcaaca
ataggaagccgctgagctcggtaagggttccttcaagtacgcctgggttcttgacaagct
caaggccgagcgtgagcgtggtatcaccatcgatattgctctctggaagttcgagactcc
tcgctactatgtcaccgtcattggtatgttgtcgctcatgcttcattctacttctcttcg
tacta--acatcactcagacgctcccggtcaccgtgatttcatcaaga
>GA_92.3
acgactcgaaacgtgcccgctaccccgctcgagaccaaaaattttgcaatatgactgtaa
ttttttttggtggggcacttaccccgccacttgagcgacgggagcgtttgccctcttaac
cattctcacaacctcaatgagtgcgtcgtcacgtgtcaagcagtcactaaccattcaaca
ataggaagccgctgagctcggtaagggttccttcaagtacgcctgggttcttgacaagct
caaggccgagcgtgagcgtggtatcaccatcgatattgctctctggaagttcgagactcc
tcgctactatgtcaccgtcattggtatgttgtcgctcatgcttcattctacttctcttcg
tacta--acatcactcagacgctcccggtcaccgtgatttcatcaaga
>GA_99.2
acgactcgaaacgtgcccgctaccccgctcgagaccaaaaattttgcaatatgactgtaa
ttttttttggtggggcacttaccccgccacttgagcgacgggagcgtttgccctcttaac
cattctcacaacctcaatgagtgcgtcgtcacgtgtcaagcagtcactaaccattcaaca
ataggaagccgctgagctcggtaagggttccttcaagtacgcctgggttcttgacaagct

```
caaggccgagcgtgagcgtggtatcaccatcgatattgctctctggaagttcgagactcc
tcgctactatgtcaccgtcattggtatgttgtcgctcatgcttcattctacttctcttcg
tacta--acatcactcagacgctcccggtcaccgtgatttcatcaaga
>NL_FOA01
acgactcgaaacgtgcccgctaccccgctcgagaccaaaaattttgcaatatgaccgtaa
-tttttttggtggggcacttaccccgccacttgagcgacgggagcgtttgccctcttaac
cattctcagaacctcaatgagtgcgtcgtcacgtgtcaagcagtcactaaccattcaaca
ataggaagccgctgagctcggtaagggttccttcaagtacgcctgggttcttgacaagct
caaggccgagcgtgagcgtggtatcaccatcgatattgctctctggaagttcgagactcc
tcgctactatgtcaccgtcattggtatgttgtcgctcatgcttcattctacttctcttcg
tacta--acatcactcagacgctcccggtcaccgtgatttcatcaaga
>NL_FOA02
acgactcgaaacgtgcccgctaccccgctcgagaccaaaaattttgcaatatgaccgtaa
-tttttttggtggggcacttaccccgccacttgagcgacgggagcgtttgccctcttaac
cattctcagaacctcaatgagtgcgtcgtcacgtgtcaagcagtcactaaccattcaaca
ataggaagccgctgagctcggtaagggttccttcaagtacgcctgggttcttgacaagct
caaggccgagcgtgagcgtggtatcaccatcgatattgctctctggaagttcgagactcc
tcgctactatgtcaccgtcattggtatgttgtcgctcatgcttcattctacttctcttcg
tacta--acatcactcagacgctcccggtcaccgtgatttcatcaaga
>NL_FOA03
acgactcgaaacgtgcccgctaccccgctcgagaccaaaaattttgcaatatgaccgtaa
-tttttttggtggggcacttaccccgccacttgagcgacgggagcgtttgccctcttaac
cattctcacaacctcaatgagtgcgtcgtcacgtgtcaagcagtcactaaccattcaaca
ataggaagccgctgagctcggtaagggttccttcaagtacgcctgggttcttgacaagct
caaggccgagcgtgagcgtggtatcaccatcgatattgctctctggaagttcgagactcc
tcgctactatgtcaccgtcattggtatgttgtcgctcatgcttcattctacttctcttcg
tacta--acatcactcagacgctcccggtcaccgtgatttcatcaaga
>NL_FOA04
acgactcgaaacgtgcccgctaccccgctcgagaccaaaaattttgcaatatgaccgtaa
ttttttttggtggggcacttaccccgccacttgagcgacgggagcgtttgccctcttaac
cattctcacaacctcaatgagtgcgtcgtcacgtgtcaagcagtcactaaccattcaaca
ataggaagccgctgagctcggtaagggttccttcaagtacgcctgggttcttgacaagct
caaggccgagcgtgagcgtggtatcaccatcgatattgctctctggaagttcgagactcc
tcgctactatgtcaccgtcattggtatgttgtcgctcatgcttcattctacttctcttcg
tacta--acatcactcagacgctcccggtcaccgtgatttcatcaaga
>NL_FOA05
acgactcgaaacgtgcccgctaccccgctcgagaccaaaaattttgcaatatgaccgtaa
ttttttttggtggggcacttaccccgccacttgagcgacgggagcgtttgccctcttaac
cattctcagaacctcaatgagtgcgtcgtcacgtgtcaagcagtcactaaccattcaaca
ataggaagccgctgagctcggtaagggttccttcaagtacgcctgggttcttgacaagct
caaggccgagcgtgagcgtggtatcaccatcgatattgctctctggaagttcgagactcc
tcgctactatgtcaccgtcattggtatgttgtcgctcatgcttcattctacttctcttcg
tacta--acatcactcagacgctcccggtcaccgtgatttcatcaaga
>SP_FOA01
acgactcgaagcgtgcccgctaccccgctcgagaccaagaatcttgcaatatgaccgtaa
-tttttttggtggggcacttaccccgccacttgagcgacgggagcgtttgccctcttaac
cattctcacaacctcaatgagtgcgtcgtcacgtgtcaagcagtcactaaccattcaaca
ataggaagccgctgagctcggtaagggttccttcaagtacgcctgggttcttgacaagct
caaggccgagcgtgagcgtggtatcaccatcgatattgctctctggaagttcgagactcc
tcgctactatgtcaccgtcattggtatgttgtcgctcatgcttcattctacttctcttcg
tacta--acatcactcagacgctcccggtcaccgtgatttcatcaaga
>SP_FOA02
acgactcgaaacgtgcccgctaccccgctcgagaccaaaaattttgcaatatgactgtaa
ttttttttggtggggcacttaccccgccacttgagcgacgggagcgtttgccctcttaac
cattctcacaacctcaatgagtgcgtcgtcacgtgtcaagcagtcactaaccattcaaca
ataggaagccgctgagctcggtaagggttccttcaagtacgcctgggttcttgacaagct
caaggccgagcgtgagcgtggtatcaccatcgatattgctctctggaagttcgagactcc
tcgctactatgtcaccgtcattggtatgttgtcgctcatgcttcattctacttctcttcg
tacta--acatcactcagacgctcccggtcaccgtgatttcatcaaga
>SP_FOA03
acgactcgaagcgtgcccgctaccccgctcgagaccaagaatcttgcaatatgaccgtaa
```

-ttttttggtggggcacttaccccgccacttgagcgacgggagcgtttgccctcttaac
cattctcacaacctcaatgagtgcgtcgtcacgtgtcaagcagtcactaaccattcaaca
ataggaagccgctgagctcggtaagggttccttcaagtacgcctgggttcttgacaagct
caaggccgagcgtgagcgtggtatcaccatcgatattgctctctggaagttcgagactcc
tcgctactatgtcaccgtcattggtatgttgtcgctcatgcttcattctacttctcttcg
tacta--acatcactcagacgctcccggtcaccgtgatttcatcaaga
>SP_FOA04
acgactcgaagcgtgcccgctaccccgctcgagaccaagaatcttgcaatatgaccgtaa
-ttttttggtggggcacttaccccgccacttgagcgacgggagcgtttgccctcttaac
cattctcacaacctcaatgagtgcgtcgtcacgtgtcaagcagtcactaaccattcaaca
ataggaagccgctgagctcggtaagggttccttcaagtacgcctgggttcttgacaagct
caaggccgagcgtgagcgtggtatcaccatcgatattgctctctggaagttcgagactcc
tcgctactatgtcaccgtcattggtatgttgtcgctcatgcttcattctacttctcttcg
tacta--acatcactcagacgctcccggtcaccgtgatttcatcaaga
>SP_FOA05
acgactcgaagcgtgcccgctaccccgctcgagaccaagaatcttgcaatatgaccgtaa
-ttttttggtggggcacttaccccgccacttgagcgacgggagcgtttgccctcttaac
cattctcacaacctcaatgagtgcgtcgtcacgtgtcaagcagtcactaaccattcaaca
ataggaagccgctgagctcggtaagggttccttcaagtacgcctgggttcttgacaagct
caaggccgagcgtgagcgtggtatcaccatcgatattgctctctggaagttcgagactcc
tcgctactatgtcaccgtcattggtatgttgtcgctcatgcttcattctacttctcttcg
tacta--acatcactcagacgctcccggtcaccgtgatttcatcaaga
>SP_FOA06
acgactcgaaacgtgcccgctaccccgctcgagaccaaaaattttgcaatatgaccgtaa
tttttttggtggggcacttaccccgccacttgagcgacgggagcgtttgccctcttaac
cattctcacaacctcaatgagtgcgtcgtcacgtgtcaagcagtcactaaccattcaaca
ataggaagccgctgagctcggtaagggttccttcaagtacgcctgggttcttgacaagct
caaggccgagcgtgagcgtggtatcaccatcgatattgctctctggaagttcgagactcc
tcgctactatgtcaccgtcattggtatgttgtcgctcatgcttcattctacttctcttcg
tacta--acatcactcagacgctcccggtcaccgtgatttcatcaaga
>SP_FOA07
acgactcgaagcgtgcccgctaccccgctcgagaccaagaatcttgcaatatgaccgtaa
-ttttttggtggggcacttaccccgccacttgagcgacgggagcgtttgccctcttaac
cattctcacaacctcaatgagtgcgtcgtcacgtgtcaagcagtcactaaccattcaaca
ataggaagccgctgagctcggtaagggttccttcaagtacgcctgggttcttgacaagct
caaggccgagcgtgagcgtggtatcaccatcgatattgctctctggaagttcgagactcc
tcgctactatgtcaccgtcattggtatgttgtcgctcatgcttcattctacttctcttcg
tacta--acatcactcagacgctcccggtcaccgtgatttcatcaaga
>SP_FOA08
acgactcgaaacgtgcccgctaccccgctcgagaccaaaaattttgcaatatgaccgtaa
-ttttttggtggggcacttaccccgccacttgagcgacgggagcgtttgccctcttaac
cattctcacaacctcaatgagtgcgtcgtcacgtgtcaagcagtcactaaccattcaaca
ataggaagccgctgagctcggtaagggttccttcaagtacgcctgggttcttgacaagct
caaggccgagcgtgagcgtggtatcaccatcgatattgctctctggaagttcgagactcc
tcgctactatgtcaccgtcattggtatgttgtcgctcatgcttcattctacttctcttcg
tacta--acatcactcagacgctcccggtcaccgtgatttcatcaaga
>SP_FOA09
acgactcgaaacgtgcccgctaccccgctcgagaccaaaaattttgcaatatgaccgtaa
-ttttttggtggggcacttaccccgccacttgagcgacgggagcgtttgccctcttaac
cattctcacaacctcaatgagtgcgtcgtcacgtgtcaagcagtcactaaccattcaaca
ataggaagccgctgagctcggtaagggttccttcaagtacgcctgggttcttgacaagct
caaggccgagcgtgagcgtggtatcaccatcgatattgctctctggaagttcgagactcc
tcgctactatgtcaccgtcattggtatgttgtcgctcatgcttcattctacttctcttcg
tacta--acatcactcagacgctcccggtcaccgtgatttcatcaaga
>SP_FOA10
acgactcgaaacgtgcccgctaccccgctcgagaccaaaaattttgcaatatgaccgtaa
tttttttggtggggcacttaccccgccacttgagcgacgggagcgtttgccctcttaac
cattctcacaacctcaatgagtgcgtcgtcacgtgtcaagcagtcactaaccattcaaca
ataggaagccgctgagctcggtaagggttccttcaagtacgcctgggttcttgacaagct
caaggccgagcgtgagcgtggtatcaccatcgatattgctctctggaagttcgagactcc
tcgctactatgtcaccgtcattggtatgttgtcgctcatgcttcattctacttctcttcg

tacta--acatcactcagacgctcccggtcaccgtgatttcatcaaga
>SP_FOA11
acgactcgaaacgtgcccgctaccccgctcgagaccaaaaattttgcaatatgaccgtaa
ttttttttggtggggcacttaccccgccacttgagcgacgggagcgtttgccctcttaac
cattctcacaacctcaatgagtgcgtcgtcacgtgtcaagcagtcactaaccattcaaca
ataggaagccgctgagctcggtaagggttccttcaagtacgcctgggttcttgacaagct
caaggccgagcgtgagcgtggtatcaccatcgatattgctctctggaagttcgagactcc
tcgctactatgtcaccgtcattggtatgttgtcgctcatgcttcattctacttctcttcg
tacta--acatcactcagacgctcccggtcaccgtgatttcatcaaga
>SP_FOA12
acgactcgaaatgtgcccgctaccccgctcgagaccaaaaattttgcaatatgaccgtaa
ttttttttggtggggcacttaccccgccacttgagcgacgggagcgtttgccctcttaac
cattctcacaacctcaatgagtgcgtcgtcacgtgtcaagcagtcactaaccattcaaca
ataggaagccgctgagctcggtaagggttccttcaagtacgcctgggttcttgacaagct
caaggccgagcgtgagcgtggtatcaccatcgatattgctctctggaagttcgagactcc
tcgctactatgtcaccgtcattggtatgttgtcgctcatgcttcattctacttctcttcg
tacta--acatcactcagacgctcccggtcaccgtgatttcatcaaga
>SP_FOA13
acgactcgaaatgtgcccgctaccccgctcgagaccaaaaattttgcaatatgaccgtaa
ttttttttggtggggcacttaccccgccacttgagcgacgggagcgtttgccctcttaac
cattctcacaacctcaatgagtgcgtcgtcacgtgtcaagcagtcactaaccattcaaca
ataggaagccgctgagctcggtaagggttccttcaagtacgcctgggttcttgacaagct
caaggccgagcgtgagcgtggtatcaccatcgatattgctctctggaagttcgagactcc
tcgctactatgtcaccgtcattggtatgttgtcgctcatgcttcattctacttctcttcg
tacta--acatcactcagacgctcccggtcaccgtgatttcatcaaga
>SP_FOA14
acgactcgaagcgtgcccgctaccccgctcgagaccaaaaattttgcaatatgaccgtaa
ttttttttggtggggcacttaccccgccacttgagcgacgggagcgtttgccctcttaac
cattctcacaacctcaatgagtgcgtcgtcacgtgtcaagcagtcactaaccattcaaca
ataggaagccgctgagctcggtaagggttccttcaagtacgcctgggttcttgacaagct
caaggccgagcgtgagcgtggtatcaccatcgatattgctctctggaagttcgagactcc
tcgctactatgtcaccgtcattggtatgttgtcgctcatgcttcattctacttctcttcg
tacta--acatcactcagacgctcccggtcaccgtgatttcatcaaga
>SP_FOA15
acgactcgaaacgtgcccgctaccccgctcgagaccaaaaattttgcaatatgactgtaa
ttttttttggtggggcacttaccccgccacttgagcgacgggagcgtttgccctcttaac
cattctcacaacctcaatgagtgcgtcgtcacgtgtcaagcagtcactaaccattcaaca
ataggaagccgctgagctcggtaagggttccttcaagtacgcctgggttcttgacaagct
caaggccgagcgtgagcgtggtatcaccatcgatattgctctctggaagttcgagactcc
tcgctactatgtcaccgtcattggtatgttgtcgctcatgcttcattctacttctcttcg
tacta--acatcactcagacgctcccggtcaccgtgatttcatcaaga
>SP_FOA16
acgactcgaaacgtgcccgctaccccgctcgagaccaaaaattttgcaatatgactgtaa
ttttttttggtggggcacttaccccgccacttgagcgacgggagcgtttgccctcttaac
cattctcacaacctcaatgagtgcgtcgtcacgtgtcaagcagtcactaaccattcaaca
ataggaagccgctgagctcggtaagggttccttcaagtacgcctgggttcttgacaagct
caaggccgagcgtgagcgtggtatcaccatcgatattgctctctggaagttcgagactcc
tcgctactatgtcaccgtcattggtatgttgtcgctcatgcttcattctacttctcttcg
tacta--acatcactcagacgctcccggtcaccgtgatttcatcaaga
>SP_FOA17
acgactcgaaacgtgcccgctaccccgctcgagaccaaaaattttgcaatatgactgtaa
ttttttttggtggggcacttaccccgccacttgagcgacgggagcgtttgccctcttaac
cattctcacaacctcaatgagtgcgtcgtcacgtgtcaagcagtcactaaccattcaaca
ataggaagccgctgagctcggtaagggttccttcaagtacgcctgggttcttgacaagct
caaggccgagcgtgagcgtggtatcaccatcgatattgctctctggaagttcgagactcc
tcgctactatgtcaccgtcattggtatgttgtcgctcatgcttcattctacttctcttcg
tacta--acatcactcagacgctcccggtcaccgtgatttcatcaaga
>SP_FOA18
acgactcgaaacgtgcccgctaccccgctcgagaccaaaaattttgcaatatgactgtaa
ttttttttggtggggcacttaccccgccacttgagcgacgggagcgtttgccctcttaac
cattctcacaacctcaatgagtgcgtcgtcacgtgtcaagcagtcactaaccattcaaca

ataggaagccgctgagctcggtaagggttccttcaagtacgcctgggttcttgacaagct
caaggccgagcgtgagcgtggtatcaccatcgatattgctctctggaagttcgagactcc
tcgctactatgtcaccgtcattggtatgttgtcgctcatgcttcattctacttctcttcg
tacta--acatcactcagacgctcccggtcaccgtgatttcatcaaga
>SP_FOA19
acgactcgaaacgtgcccgctaccccgctcgagaccaaaattttgcaatatgactgtaa
ttttttttggtggggcacttaccccgccacttgagcgacgggagcgtttgccctcttaac
cattctcacaacctcaatgagtgcgtcgtcacgtgtcaagcagtcactaaccattcaaca
ataggaagccgctgagctcggtaagggttccttcaagtacgcctgggttcttgacaagct
caaggccgagcgtgagcgtggtatcaccatcgatattgctctctggaagttcgagactcc
tcgctactatgtcaccgtcattggtatgttgtcgctcatgcttcattctacttctcttcg
tacta--acatcactcagacgctcccggtcaccgtgatttcatcaaga
>SP_FOA20
acgactcgaaacgtgcccgctaccccgctcgagaccaaaattttgcaatatgactgtaa
ttttttttggtggggcacttaccccgccacttgagcgacgggagcgtttgccctcttaac
cattctcacaacctcaatgagtgcgtcgtcacgtgtcaagcagtcactaaccattcaaca
ataggaagccgctgagctcggtaagggttccttcaagtacgcctgggttcttgacaagct
caaggccgagcgtgagcgtggtatcaccatcgatattgctctctggaagttcgagactcc
tcgctactatgtcaccgtcattggtatgttgtcgctcatgcttcattctacttctcttcg
tacta--acatcactcagacgctcccggtcaccgtgatttcatcaaga

S2: Final alignments in FASTA format (RPB1)

>fusarium_proliferatum_et1_gca_900067095
catttgcgccgaaccaatactcccattggacgagatggtaaattggccaagccccgacag
cttcacaatactcactggggtttggtgtgtcccgccgaaacgcctgagggtcaagcttgt
ggtctggtcaaaaacttgtctctgatgtgttatgtcagtgtcggctctccagccgatcct
ctcattgaattcatgatcaacagaggtatggaagtcgttgaggagtacgagccgacaaga
tatccccacgctacaaagattttcgtcaacggtagctgggttggtgttcacgccgacccc
aagcatctcgtgaatcaggttttggacacaagacgaaagtcgtacgtccagttcgaagta
tcacttgttcgtgatattcgagaccgtgaattcaagatcttctcagatgctggtcgtgtc
atgagacctgtcttcacagtccatcaggaggatgactacgagaacaacatcaccaaggga
caactagtgttgacaaaggaacatgtcaacaggctagcgcaagagcaggcagagccacct
gccaaccccgctgacaagtttggatgggatggcttgattcgcgaaggagctgtcgagtat
ctcgacgccgaggaagaagagacagccatgatttgcatgacgccagaggatctcgaactt
taccgtgagcaaaagaatgatgaagctacactcacggaagaggagaaacgggccaaggct
gaggcagagaagagggaacaggaggaggaccgcaacaagcgattgaagacaaaggtcaac
cccacaactcacatgtacacacattgcgagattcaccccagtatgattctcggtatctgt
gccagtatcattc
>fusarium_oxysporum_fo47_gca_000271705
catttgcgtcgaaccaatactcccatcggacgagatggtaaattggccaagcctcgacag
cttcacaacactcactggggtttggtgtgtcctgccgaaacacctgagggtcaagcttgt
ggtctggtcaaaaacttgtctctgatgtgttacgtcagtgtcggctctccagccgatcct
ctgattgaattcatgatcaacagaggtatggaagtcgttgaggagtacgagccgacaaga
tacccccacgctacaaagattttcgtcaacggtagctgggttggtgttcatgccgacccc
aagcatctcgtgaatcaggtcttggacacaagacgaaagtcttacgtgcagttcgaagta
tcacttgttcgtgatatccgagaccgtgaattcaagattttctcagacgctggccgtgtc
atgagacccgtctttacagttcatcaggaggatgactatgagaacaacatcaccaaggga
caactagtgttgacaaaggaccatgtcaataggctagcccaagaacaggcagagcctcct
gccaacccagcggacaagtttggatgggatggcttgatccgcgaaggagctgtcgagtat
ctcgatgctgaggaagaagagacagccatgatttgcatgacgccagaggatctcgaactt
taccgtgagcaaaagaatgatgaagctacactcacagaagaagagaaacgggccaagcaa
gaggcagagaagagagaacaagaggaggaccgcaacaagcgattgaagacaaaggtgaac
cccacaactcacatgtacacacattgtgagattcaccccagtatgattctcggtatctgt
gccagtatcattc
>GASP_2
catttgcgtcgaaccaatactcccatcggacgagatggtaaattggccaagcctcgacag
cttcacaacactcactggggtttggtgtgtcctgccgaaacacctgagggtcaagcttgt

ggtctggtcaaaaacttgtctctaatgtgttacgtcagtgtcggctctccagccgatcct
ctgattgaattcatgatcaacagaggcatggaagtcgttgaggagtacgagccgacaaga
tacccccacgctacaaagattttcgtcaacggtagctgggttggtgttcatgccgacccc
aagcatctcgtgaatcaggtcttggacacaagacgaaagtcttacgtgcagttcgaagta
tcacttgttcgtgatatccgagaccgtgaattcaagattttctcagacgctggccgtgtc
atgagacccgtctttacagttcatcaggaggatgactatgagaacaacatcaccaaggga
caactagtgttgacaaaggaccatgtcaataggctagcccaagaacaggcagagcctcct
gccaacccagcggacaagtttggatgggatggcttgatccgcgaaggagctgtcgagtat
ctcgatgctgaggaagaagagacagccatgatttgcatgacgccagaggatctcgaactt
taccgtgagcaaaagaatgatgaagctacactcacagaagaagagaaacgggccaagcaa
gaggcagagaagagagagaacaagaggaggaccgcaacaagcgattgaagacaaaggtgaac
cccacaactcacatgtacacacattgtgagattcaccccagtatgattctcggtatctgt
gccagtatcattc
>GASP_3
catttgcgtcgaaccaatactcccatcggacgagatggtaaattggccaagcctcgacag
cttcacaacactcactggggtttggtgtgtcctgccgaaacacctgagggtcaagcttgt
ggtctggtcaaaaacttgtctctaatgtgttacgtcagtgtcggctctccagccgatcct
ctgattgaattcatgatcaacagaggcatggaagtcgttgaggagtacgagccgacaaga
tacccccacgctacaaagattttcgtcaacggtagctgggttggtgttcatgccgacccc
aagcatctcgtgaatcaggtcttggacacaagacgaaagtcttacgtgcagttcgaagta
tcacttgttcgtgatatccgagaccgtgaattcaagattttctcagacgctggccgtgtc
atgagacccgtctttacagttcatcaggaggatgactatgagaacaacatcaccaaggga
caactagtgttgacaaaggaccatgtcaataggctagcccaagaacaggcagagcctcct
gccaacccagcggacaagtttggatgggatggcttgatccgcgaaggagctgtcgagtat
ctcgatgctgaggaagaagagacagccatgatttgcatgacgccagaggatctcgaactt
taccgtgagcaaaagaatgatgaagctacactcacagaagaagagaaacgggccaagcaa
gaggcagagaagagagagaacaagaggaggaccgcaacaagcgattgaagacaaaggtgaac
cccacaactcacatgtacacacattgtgagattcaccccagtatgattctcggtatctgt
gccagtatcattc
>GASP_4
catttgcgtcgaaccaatactcccatcggacgagatggtaaattggccaagcctcgacag
cttcacaacactcactggggtttggtgtgtcctgccgaaacacctgagggtcaagcttgt
ggtctggtcaaaaacttgtctctgatgtgttacgtcagtgtcggctctccagccgatcct
ctgattgaattcatgatcaacagaggcatggaagtcgttgaggagtacgagccgacaaga
tacccccacgctacaaagattttcgtcaacggtagctgggttggtgttcatgccgacccc
aagcatctcgtgaatcaggtcttggacacaagacgaaagtcttacgtgcagttcgaagta
tcacttgttcgtgatatccgagaccgtgaattcaagattttctcagacgctggccgtgtc
atgagacccgtctttacagttcatcaggaggatgactatgagaacaacatcaccaaggga
caactagtgttgacaaaggaccatgtcaataggctagcccaagaacaggcagagcctcct
gccaacccagcggacaagtttggatgggatggcttgatccgcgaaggagctgtcgagtat
ctcgatgctgaggaagaagagacagccatgatttgcatgacgccagaggatcttgaactt
taccgtgagcaaaagaatgatgaagctacactcacagaagaagagaaacgggccaagcaa
gaggcagagaagagagagaacaagaggaggaccgcaacaagcgattgaagacaaaggtgaac
cccacaactcacatgtacacacattgtgagattcaccccagtatgattctcggtatctgt
gccagtatcattc
>GASP_5
---------------------------ggacgagatggtaaattggccaagcctcgacag
cttcacaacactcactggggtttggtgtgtcctgccgaaacacctgagggtcaagcttgt
ggtctggtcaaaaacttgtctctgatgtgttacgtcagtgtcggctctccagccgatcct
ctgattgaattcatgatcaacagaggcatggaagtcgttgaggagtacgagccgacaaga
tacccccacgctacaaagattttcgtcaacggtagctgggttggtgttcatgccgaccct
aagcatctcgtgaatcaggtcttggacacaagacgaaagtcttacgtgcagttcgaagta
tcacttgttcgtgatatccgagaccgtgaattcaagattttctcagacgctggccgtgtc
atgagacccgtctttacagttcatcaggaggatgactatgagaacaacatcaccaaggga
caactagtgttgacaaaggaccatgtcaataggctagcccaagaacaggcagagcctcct
gccaacccagcggacaagtttggatgggatggcttgatccgcgaaggagctgtcgagtat
ctcgatgctgaggaagaagagacagccatgatttgcatgacgccagaggatctcgaactt
taccgtgagcaaaagaatgatgaagctacactcacagaagaagagaaacgggccaagcaa
gaggcagagaagagagagaacaagaggaggaccgcaacaagcgattgaagacaaaggtgaac
cccacaactcacatgtacacacattgtgagattcaccccagtatgattctcggtatctgt
gccagcatcattc

>GASP_9
catttgcgtcgaaccaatactcccatcggacgagatggtaaattggccaagcctcgacag
cttcacaacactcactggggtttggtgtgtcctgccgaaacacctgagggtcaagcttgt
ggtctggtcaaaaacttgtctctaatgtgttacgtcagtgtcggctctccagccgatcct
ctgattgaattcatgatcaacagaggcatggaagtcgttgaggagtacgagccgacaaga
tacccccacgctacaaagattttcgtcaacggtagctgggttggtgttcatgccgacccc
aagcatctcgtgaatcaggtcttggacacaagacgaaagtcttacgtgcagttcgaagta
tcacttgttcgtgatatccgagaccgtgaattcaagattttctcagacgctggccgtgtc
atgagacccgtctttacagttcatcaggaggatgactatgagaacaacatcaccaaggga
caactagtgttgacaaaggaccatgtcaataggctagcccaagaacaggcagagcctcct
gccaacccagcggacaagtttggatgggatggcttgatccgcgaaggagctgtcgagtat
ctcgatgctgaggaagaagagacagccatgatttgcatgacgccagaggatctcgaactt
taccgtgagcaaaagaatgatgaagctacactcacagaagaagagaaacgggccaagcaa
gaggcagagaagagagagaacaagaggaggaccgcaacaagcgattgaagacaaaggtgaac
cccacaactcacatgtacacacattgtgagattcaccccagtatgattctcggtatctgt
gccagtatcattc
>GA_1.2
catttgcgtcgaaccaatactcccatcggacgagatggtaaattggccaagcctcgacag
cttcacaacactcactggggtttggtgtgtcctgccgaaacacctgagggtcaagcttgt
ggtctggtcaaaaacttgtctctaatgtgttacgtcagtgtcggctctccagccgatcct
ctgattgaattcatgatcaacagaggcatggaagtcgttgaggagtacgagccgacaaga
tacccccacgctacaaagattttcgtcaacggtagctgggttggtgttcatgccgacccc
aagcatctcgtgaatcaggtcttggacacaagacgaaagtcttacgtgcagttcgaagta
tcacttgttcgtgatatccgagaccgtgaattcaagattttctcagacgctggccgtgtc
atgagacccgtctttacagttcatcaggaggatgactatgagaacaacatcaccaaggga
caactagtgttgacaaaggaccatgtcaataggctagcccaagaacaggcagagcctcct
gccaacccagcggacaagtttggatgggatggcttgatccgcgaaggagctgtcgagtat
ctcgatgctgaggaagaagagacagccatgatttgcatgacgccagaggatctcgaactt
taccgtgagcaaaagaatgatgaagctacactcacagaagaagagaaacgggccaagcaa
gaggcagagaagagagagaacaagaggaggaccgcaacaagcgattgaagacaaaggtgaac
cccacaactcacatgtacacacattgtgagattcaccccagtatgattctcggtatctgt
gccagtatcattc
>GA_1.3
catttgcgtcgaaccaatactcccatcggacgagatggtaaattggccaagcctcgacag
cttcacaacactcactggggtttggtgtgtcctgccgaaacacctgagggtcaagcttgt
ggtctggtcaaaaacttgtctctaatgtgttacgtcagtgtcggctctccagccgatcct
ctgattgaattcatgatcaacagaggcatggaagtcgttgaggagtacgagccgacaaga
tacccccacgctacaaagattttcgtcaacggtagctgggttggtgttcatgccgacccc
aagcatctcgtgaatcaggtcttggacacaagacgaaagtcttacgtgcagttcgaagta
tcacttgttcgtgatatccgagaccgtgaattcaagattttctcagacgctggccgtgtc
atgagacccgtctttacagttcatcaggaggatgactatgagaacaacatcaccaaggga
caactagtgttgacaaaggaccatgtcaataggctagcccaagaacaggcagagcctcct
gccaacccagcggacaagtttggatgggatggcttgatccgcgaaggagctgtcgagtat
ctcgatgctgaggaagaagagacagccatgatttgcatgacgccagaggatctcgaactt
taccgtgagcaaaagaatgatgaagctacactcacagaagaagagaaacgggccaagcaa
gaggcagagaagagagagaacaagaggaggaccgcaacaagcgattgaagacaaaggtgaac
cccacaactcacatgtacacacattgtgagattcaccccagtatgattctcggtatctgt
gccagtatcattc
>GA_1.4
catttgcgtcnaaccaatactcccatcggacgagatggtaaattggccaagcctcgacag
cttcacaacactcactggggtttggtgtgtcctgccgaaacacctgagggtcaagcttgt
ggtctggtcaaaaacttgtctctaatgtgttacgtcagtgtcggctctccagccgatcct
ctgattgaattcatgatcaacagaggcatggaagtcgttgaggagtacgagccgacaaga
tacccccacgctacaaagattttcgtcaacggtagctgggttggtgttcatgccgacccc
aagcatctcgtgaatcaggtcttggacacaagacgaaagtcttacgtgcagttcgaagta
tcacttgttcgtgatatccgagaccgtgaattcaagattttctcagacgctggccgtgtc
atgagacccgtctttacagttcatcaggaggatgactatgagaacaacatcaccaaggga
caactagtgttgacaaaggaccatgtcaataggctagcccaagaacaggcagagcctcct
gccaacccagcggacaagtttggatgggatggcttgatccgcgaaggagctgtcgagtat
ctcgatgctgaggaagaagagacagccatgatttgcatgacgccagaggatctcgaactt
taccgtgagcaaaagaatgatgaagctacactcacagaagaagagaaacgggccaagcaa

gaggcagagaagagagaacaagaggaggaccgcaacaagcgattgaagacaaaggtgaac
cccacaactcacatgtacacacattgtgagattcaccccagtatgattctcggtatctgt
gccagtatcattc
>GA_10
catttgcgtcgaaccaatactcccatcggacgagatggtaaattggccaagcctcgacag
cttcacaacactcactggggtttggtgtgtcctgccgaaacacctgagggtcaagcttgt
ggtctggtcaaaaacttgtctctaatgtgttacgtcagtgtcggctctccagccgatcct
ctgattgaattcatgatcaacagaggcatggaagtcgttgaggagtacgagccgacaaga
tacccccacgctacaaagattttcgtcaacggtagctgggttggtgttcatgccgacccc
aagcatctcgtgaatcaggtcttggacacaagacgaaagtcttacgtgcagttcgaagta
tcacttgttcgtgatatccgagaccgtgaattcaagattttctcagacgctggccgtgtc
atgagacccgtctttacagttcatcaggaggatgactatgagaacaacatcaccaaggga
caactagtgttgacaaaggaccatgtcaataggctagcccaagaacaggcagagcctcct
gccaacccagcggacaagtttggatgggatggcttgatccgcgaaggagctgtcgagtat
ctcgatgctgaggaagaagagacagccatgatttgcatgacgccagaggatctcgaactt
taccgtgagcaaaagaatgatgaagctacactcacagaagaagagaaacgggccaagcaa
gaggcagagaagagagaacaagaggaggaccgcaacaagcgattgaagacaaaggtgaac
cccacaactcacatgtacacacattgtgagattcaccccagtatgattctcggtatctgt
gccagtatcattc
>GA_3.2
catttgcgtcgaaccaatactcccatcggacgagatggtaaattggccaagcctcgacag
cttcacaacactcactggggtttggtgtgtcctgccgaaacacctgagggtcaagcttgt
ggtctggtcaaaaacttgtctctaatgtgttacgtcagtgtcggctctccagccgatcct
ctgattgaattcatgatcaacagaggcatggaagtcgttgaggagtacgagccgacaaga
tacccccacgctacaaagattttcgtcaacggtagctgggttggtgttcatgccgacccc
aagcatctcgtgaatcaggtcttggacacaagacgaaagtcttacgtgcagttcgaagta
tcacttgttcgtgatatccgagaccgtgaattcaagattttctcagacgctggccgtgtc
atgagacccgtctttacagttcatcaggaggatgactatgagaacaacatcaccaaggga
caactagtgttgacaaaggaccatgtcaataggctagcccaagaacaggcagagcctcct
gccaacccagcggacaagtttggatgggatggcttgatccgcgaaggagctgtcgagtat
ctcgatgctgaggaagaagagacagccatgatttgcatgacgccagaggatctcgaactt
taccgtgagcaaaagaatgatgaagctacactcacagaagaagagaaacgggccaagcaa
gaggcagagaagagagaacaagaggaggaccgcaacaagcgattgaagacaaaggtgaac
cccacaactcacatgtacacacattgtgagattcaccccagtatgattctcggtatctgt
gccagtatcattc
>GA_3.3
catttgcgtcgaaccaatactcccatcggacgagatggtaaattggccaagcctcgacag
cttcacaacactcactggggtttggtgtgtcctgccgaaacacctgagggtcaagcttgt
ggtctggtcaaaaacttgtctctaatgtgttacgtcagtgtcggctctccagccgatcct
ctgattgaattcatgatcaacagaggcatggaagtcgttgaggagtacgagccgacaaga
tacccccacgctacaaagattttcgtcaacggtagctgggttggtgttcatgccgacccc
aagcatctcgtgaatcaggtcttggacacaagacgaaagtcttacgtgcagttcgaagta
tcacttgttcgtgatatccgagaccgtgaattcaagattttctcagacgctggccgtgtc
atgagacccgtctttacagttcatcaggaggatgactatgagaacaacatcaccaaggga
caactagtgttgacaaaggaccatgtcaataggctagcccaagaacaggcagagcctcct
gccaacccagcggacaagtttggatgggatggcttgatccgcgaaggagctgtcgagtat
ctcgatgctgaggaagaagagacagccatgatttgcatgacgccagaggatctcgaactt
taccgtgagcaaaagaatgatgaagctacactcacagaagaagagaaacgggccaagcaa
gaggcagagaagagagaacaagaggaggaccgcaacaagcgattgaagacaaaggtgaac
cccacaactcacatgtacacacattgtgagattcaccccagtatgattctcggtatctgt
gccagtatcattc
>GA_3.5
catttgcgtcnaaccaatactcccatcggacgagatggtaaattggccaagcctcgacag
cttcacaacactcactggggtttggtgtgtcctgccgaaacacctgagggtcaagcttgt
ggtctggtcaaaaacttgtctctaatgtgttacgtcagtgtcggctctccagccgatcct
ctgattgaattcatgatcaacagaggcatggaagtcgttgaggagtacgagccgacaaga
tacccccacgctacaaagattttcgtcaacggtagctgggttggtgttcatgccgacccc
aagcatctcgtgaatcaggtcttggacacaagacgaaagtcttacgtgcagttcgaagta
tcacttgttcgtgatatccgagaccgtgaattcaagattttctcagacgctggccgtgtc
atgagacccgtctttacagttcatcaggaggatgactatgagaacaacatcaccaaggga
caactagtgttgacaaaggaccatgtcaataggctagcccaagaacaggcagagcctcct

gccaacccagcggacaagtttggatgggatggcttgatccgcgaaggagctgtcgagtat
ctcgatgctgaggaagaagagacagccatgatttgcatgacgccagaggatctcgaactt
taccgtgagcaaaagaatgatgaagctacactcacagaagaagagaaacgggccaagcaa
gaggcagagaagagagagaacaagaggaggaccgcaacaagcgattgaagacaaaggtgaac
cccacaactcacatgtacacacattgtgagattcaccccagtatgattctcggtatctgt
gccagtatcattc
>GA_3.6
catttgcgtcgaaccaatactcccatcggacgagatggtaaattggccaagcctcgacag
cttcacaacactcactggggtttggtgtgtcctgccgaaacacctgagggtcaagcttgt
ggtctggtcaaaaacttgtctctaatgtgttacgtcagtgtcggctctccagccgatcct
ctgattgaattcatgatcaacagaggcatggaagttgttgaggagtacgagccgacaaga
taccccccacgctacaaagattttcgtcaacggtagctgggttggtgttcatgccgacccc
aagcatctcgtgaatcaggtcttggacacaagacgaaagtcttacgtgcagttcgaagta
tcacttgttcgtgatatccgagaccgtgaattcaagattttctcagacgctggccgtgtc
atgcgacccgtctttacagttcatcaggaggatgactatgagaacaacatcaccaaggga
caactagtgttgacaaaggaccatgtcaataggctagcccaagaacaggcagagcctcct
gccaacccagcggacaagtttggatgggatggcttgatccgcgaaggagctgtcgagtat
ctcgatgctgaggaagaagagacagccatgatttgcatgacgccagaggatctcgaactt
taccgtgagcaaaagaatgatgaagctacactcacagaagaagagaaacgggccaagcaa
gaggcagagaagagagagaacaagaggaggaccgcaacaagcgattgaagacaaaggtgaac
cccacaactcacatgtacacacattgtgagattcaccccagtatgattctcggtatctgt
gccagtatca---
>GA_3.7
catttgcgtcgaaccaatactcccatcggacgagatggtaaattggccaagcctcgacag
cttcacaacactcactggggtttggtgtgtcctgccgaaacacctgagggtcaagcttgt
ggtctggtcaaaaacttgtctctaatgtgttacgtcagtgtcggctctccagccgatcct
ctgattgaattcatgatcaacagaggcatggaagtcgttgaggagtacgagccgacaaga
taccccccacgctacaaagattttcgtcaacggtagctgggttggtgttcatgccgacccc
aagcatctcgtgaatcaggtcttggacacaagacgaaagtcttacgtgcagttcgaagta
tcacttgttcgtgatatccgagaccgtgaattcaagattttctcagacgctggccgtgtc
atgagacccgtctttacagttcatcaggaggatgactatgagaacaacatcaccaaggga
caactagtgttgacaaaggaccatgtcaataggctagcccaagaacaggcagagcctcct
gccaacccagcggacaagtttggatgggatggcttgatccgcgaaggagctgtcgagtat
ctcgatgctgaggaagaagagacagccatgatttgcatgacgccagaggatctcgaactt
taccgtgagcaaaagaatgatgaagctacactcacagaagaagagaaacgggccaagcaa
gaggcagagaagagagagaacaagaggaggaccgcaacaagcgattgaagacaaaggtgaac
cccacaactcacatgtacacacattgtgagattcaccccagtatgattctcggtatctgt
gccagtatcattc
>GA_4.1
catttgcgtcgaaccaatactcccatcggacgagatggtaaattggccaagcctcgacag
cttcacaacactcactggggtttggtgtgtcctgccgaaacacctgagggtcaagcttgt
ggtctggtcaaaaacttgtctctaatgtgttacgtcagtgtcggctctccagccgatcct
ctgattgaattcatgatcaacagaggcatggaagtcgttgaggagtacgagccgacaaga
taccccccacgctacaaagattttcgtcaacggtagctgggttggtgttcatgccgacccc
aagcatctcgtgaatcaggtcttggacacaagacgaaagtcttacgtgcagttcgaagta
tcacttgttcgtgatatccgagaccgtgaattcaagattttctcagacgctggccgtgtc
atgagacccgtctttacagttcatcaggaggatgactatgagaacaacatcaccaaggga
caactagtgttgacaaaggaccatgtcaataggctagcccaagaacaggcagagcctcct
gccaacccagcggacaagtttggatgggatggcttgatccgcgaaggagctgtcgagtat
ctcgatgctgaggaagaagagacagccatgatttgcatgacgccagaggatctcgaactt
taccgtgagcaaaagaatgatgaagctacactcacagaagaagagaaacgggccaagcaa
gaggcagagaagagagagaacaagaggaggaccgcaacaagcgattgaagacaaaggtgaac
cccacaactcacatgtacacacattgtgagattcaccccagtatgattctcggtatctgt
gccagtatcattc
>GA_4.2
catttgcgtcgaaccaatactcccatcggacgagatggtaaattggccaagcctcgacag
cttcacaacactcactggggtttggtgtgtcctgccgaaacacctgagggtcaagcttgt
ggtctggtcaaaaacttgtctctaatgtgttacgtcagtgtcggctctccagccgatcct
ctgattgaattcatgatcaacagaggcatggaagtcgttgaggagtacgagccgacaaga
taccccccacgctacaaagattttcgtcaacggtagctgggttggtgttcatgccgacccc
aagcatctcgtgaatcaggtcttggacacaagacgaaagtcttacgtgcagttcgaagta

tcacttgttcgtgatatccgagaccgtgaattcaagattttctcagacgctggccgtgtc
atgagacccgtctttacagttcatcaggaggatgactatgagaacaacatcaccaaggga
caactagtgttgacaaaggaccatgtcaataggctagcccaagaacaggcagagcctcct
gccaacccagcggacaagtttggatgggatggcttgatccgcgaaggagctgtcgagtat
ctcgatgctgaggaagaagagacagccatgatttgcatgacgccagaggatctcgaactt
taccgtgagcaaaagaatgatgaagctacactcacagaagaagagaaacgggccaagcaa
gaggcagagaagagagagaacaagaggaggaccgcaacaagcgattgaagacaaaggtgaac
cccacaactcacatgtacacacattgtgagattcaccccagtatgattctcggtatctgt
gccagtatcattc
>GA_4.3
catttgcgtcgaaccaatactcccatcggacgagatggtaaattggccaagcctcgacag
cttcacaacactcactggggtttggtgtgtcctgccgaaacacctgagggtcaagcttgt
ggtctggtcaaaaacttgtctctaatgtgttacgtcagtgtcggctctccagccgatcct
ctgattgaattcatgatcaacagaggcatggaagtcgttgaggagtacgagccgacaaga
tacccccacgctacaaagattttcgtcaacggtagctgggttggtgttcatgccgacccc
aagcatctcgtgaatcaggtcttggacacaagacgaaagtcttacgtgcagttcgaagta
tcacttgttcgtgatatccgagaccgtgaattcaagattttctcagacgctggccgtgtc
atgagacccgtctttacagttcatcaggaggatgactatgagaacaacatcaccaaggga
caactagtgttgacaaaggaccatgtcaataggctagcccaagaacaggcagagcctcct
gccaacccagcggacaagtttggatgggatggcttgatccgcgaaggagctgtcgagtat
ctcgatgctgaggaagaagagacagccatgatttgcatgacgccagaggatctcgaactt
taccgtgagcaaaagaatgatgaagctacactcacagaagaagagaaacgggccaagcaa
gaggcagagaagagagagaacaagaggaggaccgcaacaagcgattgaagacaaaggtgaac
cccacaactcacatgtacacacattgtgagattcaccccagtatgattctcggtatctgt
gccagtatcattc
>GA_5.3
catttgcgtcgaaccaatactcccatcggacgagatggtaaattggccaagcctcgacag
cttcacaacactcactggggtttggtgtgtcctgccgaaacacctgagggtcaagcttgt
ggtctggtcaaaaacttgtctctaatgtgttacgtcagtgtcggctctccagccgatcct
ctgattgaattcatgatcaacagaggcatggaagtcgttgaggagtacgagccgacaaga
tacccccacgctacaaagattttcgtcaacggtagctgggttggtgttcatgccgacccc
aagcatctcgtgaatcaggtcttggacacaagacgaaagtcttacgtgcagttcgaagta
tcacttgttcgtgatatccgagaccgtgaattcaagattttctcagacgctggccgtgtc
atgagacccgtctttacagttcatcaggaggatgactatgagaacaacatcaccaaggga
caactagtgttgacaaaggaccatgtcaataggctagcccaagaacaggcagagcctcct
gccaacccagcggacaagtttggatgggatggcttgatccgcgaaggagctgtcgagtat
ctcgatgctgaggaagaagagacagccatgatttgcatgacgccagaggatctcgaactt
taccgtgagcaaaagaatgatgaagctacactcacagaagaagagaaacgggccaagcaa
gaggcagagaagagagagaacaagaggaggaccgcaacaagcgattgaagacaaaggtgaac
cccacaactcacatgtacacacattgtgagattcaccccagtatgattctcggtatctgt
gccagtatcattc
>GA_5.4
catttgcgtcgaaccaatactcccatcggacgagatggtaaattggccaagcctcgacag
cttcacaacactcactggggtttggtgtgtcctgccgaaacacctgagggtcaagcttgt
ggtctggtcaaaaacttgtctctaatgtgttacgtcagtgtcggctctccagccgatcct
ctgattgaattcatgatcaacagaggcatggaagtcgttgaggagtacgagccgacaaga
tacccccacgctacaaagattttcgtcaacggtagctgggttggtgttcatgccgacccc
aagcatctcgtgaatcaggtcttggacacaagacgaaagtcttacgtgcagttcgaagta
tcacttgttcgtgatatccgagaccgtgaattcaagattttctcagacgctggccgtgtc
atgagacccgtctttacagttcatcaggaggatgactatgagaacaacatcaccaaggga
caactagtgttgacaaaggaccatgtcaataggctagcccaagaacaggcagagcctcct
gccaacccagcggacaagtttggatgggatggcttgatccgcgaaggagctgtcgagtat
ctcgatgctgaggaagaagagacagccatgatttgcatgacgccagaggatctcgaactt
taccgtgagcaaaagaatgatgaagctacactcacagaagaagagaaacgggccaagcaa
gaggcagagaagagagagaacaagaggaggaccgcaacaagcgattgaagacaaaggtgaac
cccacaactcacatgtacacacattgtgagattcaccccagtatgattctcggtatctgt
gccagtatcattc
>GA_92.3
catttgcgtcgaaccaatactcccatcggacgagatggtaaattggccaagcctcgacag
cttcacaacactcactggggtttggtgtgtcctgccgaaacacctgagggtcaagcttgt
ggtctggtcaaaaacttgtctctaatgtgttacgtcagtgtcggctctccagccgatcct

ctgattgaattcatgatcaacagaggcatggaagtcgttgaggagtacgagccgacaaga
tacccccacgctacaaagattttcgtcaacggtagctgggttggtgttcatgccgacccc
aagcatctcgtgaatcaggtcttggacacaagacgaaagtcttacgtgcagttcgaagta
tcacttgttcgtgatatccgagaccgtgaattcaagattttctcagacgctggccgtgtc
atgagacccgtctttacagttcatcaggaggatgactatgagaacaacatcaccaaggga
caactagtgttgacaaaggaccatgtcaataggctagcccaagaacaggcagagcctcct
gccaacccagcggacaagtttggatgggatggcttgatccgcgaaggagctgtcgagtat
ctcgatgctgaggaagaagagacagccatgatttgcatgacgccagaggatctcgaactt
taccgtgagcaaaagaatgatgaagctacactcacagaagaagagaaacgggccaagcaa
gaggcagagaagagagaacaagaggaggaccgcaacaagcgattgaagacaaaggtgaac
cccacaactcacatgtacacacattgtgagattcaccccagtatgattctcggtatctgt
gccagtatcattc
>GA_99.2
catttgcgtcnaaccaatactcccatcggacgagatggtaaattggccaagcctcgacag
cttcacaacactcactggggtttggtgtgtcctgccgaaacacctgagggtcaagcttgt
ggtctggtcaaaaacttgtctctaatgtgttacgtcagtgtcggctctccagccgatcct
ctgattgaattcatgatcaacagaggcatggaagtcgttgaggagtacgagccgacaaga
tacccccacgctacaaagattttcgtcaacggtagctgggttggtgttcatgccgacccc
aagcatctcgtgaatcaggtcttggacacaagacgaaagtcttacgtgcagttcgaagta
tcacttgttcgtgatatccgagaccgtgaattcaagattttctcagacgctggccgtgtc
atgagacccgtctttacagttcatcaggaggatgactatgagaacaacatcaccaaggga
caactagtgttgacaaaggaccatgtcaataggctagcccaagaacaggcagagcctcct
gccaacccagcggacaagtttggatgggatggcttgatccgcgaaggagctgtcgagtat
ctcgatgctgaggaagaagagacagccatgatttgcatgacgccagaggatctcgaactt
taccgtgagcaaaagaatgatgaagctacactcacagaagaagagaaacgggccaagcaa
gaggcagagaagagagaacaagaggaggaccgcaacaagcgattgaagacaaaggtgaac
cccacaactcacatgtacacacattgtgagattcaccccagtatgattctcggtatctgt
gccagtatcattc
>NL_FOA01
catttgcgtcgaaccaatactcccatcggacgagatggtaaattggccaagcctcgacag
cttcacaacactcactggggtttggtgtgtcctgccgaaacacccgagggtcaagcttgt
ggtctggtcaaaaacttgtctctgatgtgttacgtcagtgttggctctccggccgatcct
ctgattgaattcatgatcaacagaggcatggaagtcgttgaggagtacgagccgacaaga
tacccccacgctacaaagattttcgtcaacggtagctgggttggtgttcatgccgacccc
aagcatctcgtgaatcaggtcttggacacaaggcgaaagtcttacgtgcagttcgaagta
tcacttgttcgtgatatccgagaccgtgaattcaagattttctcagacgctggccgtgtc
atgagacccgtctttacagttcatcaggaggatgactatgagaacaacatcaccaaggga
caactagtgttgacaaaggaccatgtcaataggctagcccaagagcaggcagagcctcct
gccaacccagcggacaagtttggatgggatggcttgatccgcgaaggagctgtcgagtat
ctcgatgctgaggaagaagagacagccatgatttgcatgacgccagaggatctcgaactt
taccgtgagcaaaagaatgatgaagctacactcacagaagaagagaaacgggccaagcaa
gaggcagagaagagggaacaagaggaggaccgcaacaagcgattgaagacaaaggtgaac
cccacaactcacatgtacacacattgtgagattcaccccagtatgattctcggtatctgt
gccagtatcattc
>NL_FOA02
catttgcgtcgaaccaatactcccatcggacgagatggtaaattggccaagcctcgacag
cttcacaacactcactggggtttggtgtgtcctgccgaaacacccgagggtcaagcttgt
ggtctggtcaaaaacttgtctctgatgtgttacgtcagtgttggctctccggccgatcct
ctgattgaattcatgatcaacagaggcatggaagtcgttgaggagtacgagccgacaaga
tacccccacgctacaaagattttcgtcaacggtagctgggttggtgttcatgccgacccc
aagcatctcgtgaatcaggtcttggacacaaggcgaaagtcttacgtgcagttcgaagta
tcacttgttcgtgatatccgagaccgtgaattcaagattttctcagacgctggccgtgtc
atgagacccgtctttacagttcatcaggaggatgactatgagaacaacatcaccaaggga
caactagtgttgacaaaggaccatgtcaataggctagcccaagagcaggcagagcctcct
gccaacccagcggacaagtttggatgggatggcttgatccgcgaaggagctgtcgagtat
ctcgatgctgaggaagaagagacagccatgatttgcatgacgccagaggatctcgaactt
taccgtgagcaaaagaatgatgaagctacactcacagaagaagagaaacgggccaagcaa
gaggcagagaagagggaacaagaggaggaccgcaacaagcgattgaagacaaaggtgaac
cccacaactcacatgtacacacattgtgagattcaccccagtatgattctcggtatctgt
gccagtatcattc
>NL_FOA03

catttgcgtcgaaccaatactcccatcggacgagatggtaaattggccaagcctcgacag
cttcacaacactcactggggtttggtgtgtcctgccgaaacacctgagggtcaagcttgt
ggtctggtcaaaaacttgtctctaatgtgttacgtcagtgtcggctctccagccgatcct
ctgattgaattcatgatcaacagaggcatggaagtcgttgaggagtacgagccgacaaga
tacccccacgctacaaagattttcgtcaacggtagctgggttggtgttcatgccgacccc
aagcatctcgtgaatcaggtcttggacacaagacgaaagtcttacgtgcagttcgaagta
tcacttgttcgtgatatccgagaccgtgaattcaagattttctcagacgctggccgtgtc
atgagacccgtctttacagttcatcaggaggatgactatgagaacaacatcaccaaggga
caactagtgttgacaaaggaccatgtcaataggctagcccaagaacaggcagagcctcct
gccaacccagcggacaagtttggatgggatggcttgatccgcgaaggagctgtcgagtat
ctcgatgctgaggaagaagagacagccatgatttgcatgacgccagaggatctcgaactt
taccgtgagcaaaagaatgatgaagctacactcacagaagaagagaaacggccaagcaa
gaggcagagaagagagagaacaagaggaggaccgcaacaagcgattgaagacaaaggtgaac
cccacaactcacatgtacacacattgtgagattcaccccagtatgattctcggtatctgt
gccagtatcattc
>NL_FOA04
catttgcgtcgaaccaatactcccatcggacgagatggtaaattggccaagcctcgacag
cttcacaacactcactggggtttggtgtgtcctgccgaaacacctgagggtcaagcttgt
ggtctggtcaaaaacttgtctctaatgtgttacgtcagtgtcggctctccagccgatcct
ctgattgaattcatgatcaacagaggcatggaagtcgttgaggagtacgagccgacaaga
tacccccacgctacaaagattttcgtcaacggtagctgggttggtgttcatgccgacccc
aagcatctcgtgaatcaggtcttggacacaagacgaaagtcttacgtgcagttcgaagta
tcacttgttcgtgatatccgagaccgtgaattcaagattttctcagacgctggccgtgtc
atgagacccgtctttacagttcatcaggaggatgactatgagaacaacatcaccaaggga
caactagtgttgacaaaggaccatgtcaataggctagcccaagaacaggcagagcctcct
gccaacccagcggacaagtttggatgggatggcttgatccgcgaaggagctgtcgagtat
ctcgatgctgaggaagaagagacagccatgatttgcatgacgccagaggatctcgaactt
taccgtgagcaaaagaatgatgaagctacactcacagaagaagagaaacggccaagcaa
gaggcagagaagagagagaacaagaggaggaccgcaacaagcgattgaagacaaaggtgaac
cccacaactcacatgtacacacattgtgagattcaccccagtatgattctcggtatctgt
gccagtatcattc
>NL_FOA05
catttgcgtcgaaccaatactcccatcggacgagatggtaaattggccaagcctcgacag
cttcacaacactcactggggtttggtgtgtcctgccgaaacacccgagggtcaagcttgt
ggtctggtcaaaaacttgtctctgatgtgttacgtcagtgttggctctccggccgatcct
ctgattgaattcatgatcaacagaggcatggaagtcgttgaggagtacgagccgacaaga
tacccccacgctacaaagattttcgtcaacggtagctgggttggtgttcatgccgacccc
aagcatctcgtgaatcaggtcttggacacaaggcgaaagtcttacgtgcagttcgaagta
tcacttgttcgtgatatccgagaccgtgaattcaagattttctcagacgctggccgtgtc
atgagacccgtctttacagttcatcaggaggatgactatgagaacaacatcaccaaggga
caactagtgttgacaaaggaccatgtcaataggctagcccaagagcaggcagagcctcct
gccaacccagcggacaagtttggatgggatggcttgatccgcgaaggagctgtcgagtat
ctcgatgctgaggaagaagagacagccatgatttgcatgacgccagaggatctcgaactt
taccgtgagcaaaagaatgatgaagctacactcacagaagaagagaaacggccaagcaa
gaggcagagaagagggaacaagaggaggaccgcaacaagcgattgaagacaaaggtgaac
cccacaactcacatgtacacacattgtgagattcaccccagtatgattctcggtatctgt
gccagtatcattc
>SP_FOA01
catttgcgtcgaaccaatactcccatcggacgagatggtaaattggccaagcctcgacag
cttcacaacactcactggggtttggtgtgtcctgccgaaacacctgagggtcaagcttgt
ggtctggtcaaaaacttgtctctaatgtgttacgtcagtgtcggctctccagccgatcct
ctgattgaattcatgatcaacagaggcatggaagtcgttgaggagtacgagccgacaaga
tacccccacgctacaaagattttcgtcaacggtagctgggttggtgttcatgccgacccc
aagcatctcgtgaatcaggtcttggacacaagacgaaagtcttacgtgcagttcgaagta
tcacttgttcgtgatatccgagaccgtgaattcaagattttctcagacgctggccgtgtc
atgagacccgtctttacagttcatcaggaggatgactatgagaacaacatcaccaaggga
caactagtgttgacaaaggaccatgtcaataggctagcccaagaacaggcagagcctcct
gccaacccagcggacaagtttggatgggatggcttgatccgcgaaggagctgtcgagtat
ctcgatgctgaggaagaagagacagccatgatttgcatgacgccagaggatctcgaactt
taccgtgagcaaaagaatgatgaagctacactcacagaagaagagaaacggccaagcaa
gaggcagagaagagagagaacaagaggaggaccgcaacaagcgattgaagacaaaggtgaac

cccacaactcacatgtacacacattgtgagattcaccccagtatgattctcggtatctgt
gccagtatcattc
>SP_FOA02
catttgcgtcgaaccaatactcccatcggacgagatggtaaattggccaagcctcgacag
cttcacaacactcactggggtttggtgtgtcctgccgaaacacctgagggtcaagcttgt
ggtctggtcaaaaacttgtctctaatgtgttacgtcagtgtcggctctccagccgatcct
ctgattgaattcatgatcaacagaggcatggaagtcgttgaggagtacgagccgacaaga
tacccccacgctacaaagattttcgtcaacggtagctgggttggtgttcatgccgacccc
aagcatctcgtgaatcaggtcttggacacaagacgaaagtcttacgtgcagttcgaagta
tcacttgttcgtgatatccgagaccgtgaattcaagattttctcagacgctggccgtgtc
atgagacccgtctttacagttcatcaggaggatgactatgagaacaacatcaccaaggga
caactagtgttgacaaaggaccatgtcaataggctagcccaagaacaggcagagcctcct
gccaacccagcggacaagtttggatgggatggcttgatccgcgaaggagctgtcgagtat
ctcgatgctgaggaagaagagacagccatgatttgcatgacgccagaggatctcgaactt
taccgtgagcaaaagaatgatgaagctacactcacagaagaagagaaacggccaagcaa
gaggcagagaagagagagaacaagaggaggaccgcaacaagcgattgaagacaaaggtgaac
cccacaactcacatgtacacacattgtgagattcaccccagtatgattctcggtatctgt
gccagtatcattc
>SP_FOA03
catttgcgtcgaaccaatactcccatcggacgagatggtaaattggccaagcctcgacag
cttcacaacactcactggggtttggtgtgtcctgccgaaacacctgagggtcaagcttgt
ggtctggtcaaaaacttgtctctaatgtgttacgtcagtgtcggctctccagccgatcct
ctgattgaattcatgatcaacagaggcatggaagtcgttgaggagtacgagccgacaaga
tacccccacgctacaaagattttcgtcaacggtagctgggttggtgttcatgccgacccc
aagcatctcgtgaatcaggtcttggacacaagacgaaagtcttacgtgcagttcgaagta
tcacttgttcgtgatatccgagaccgtgaattcaagattttctcagacgctggccgtgtc
atgagacccgtctttacagttcatcaggaggatgactatgagaacaacatcaccaaggga
caactagtgttgacaaaggaccatgtcaataggctagcccaagaacaggcagagcctcct
gccaacccagcggacaagtttggatgggatggcttgatccgcgaaggagctgtcgagtat
ctcgatgctgaggaagaagagacagccatgatttgcatgacgccagaggatctcgaactt
taccgtgagcaaaagaatgatgaagctacactcacagaagaagagaaacggccaagcaa
gaggcagagaagagagagaacaagaggaggaccgcaacaagcgattgaagacaaaggtgaac
cccacaactcacatgtacacacattgtgagattcaccccagtatgattctcggtatctgt
gccagtatcattc
>SP_FOA04
catttgcgtcgaaccaatactcccatcggacgagatggtaaattggccaagcctcgacag
cttcacaacactcactggggtttggtgtgtcctgccgaaacacctgagggtcaagcttgt
ggtctggtcaaaaacttgtctctaatgtgttacgtcagtgtcggctctccagccgatcct
ctgattgaattcatgatcaacagaggcatggaagtcgttgaggagtacgagccgacaaga
tacccccacgctacaaagattttcgtcaacggtagctgggttggtgttcatgccgacccc
aagcatctcgtgaatcaggtcttggacacaagacgaaagtcttacgtgcagttcgaagta
tcacttgttcgtgatatccgagaccgtgaattcaagattttctcagacgctggccgtgtc
atgagacccgtctttacagttcatcaggaggatgactatgagaacaacatcaccaaggga
caactagtgttgacaaaggaccatgtcaataggctagcccaagaacaggcagagcctcct
gccaacccagcggacaagtttggatgggatggcttgatccgcgaaggagctgtcgagtat
ctcgatgctgaggaagaagagacagccatgatttgcatgacgccagaggatctcgaactt
taccgtgagcaaaagaatgatgaagctacactcacagaagaagagaaacggccaagcaa
gaggcagagaagagagagaacaagaggaggaccgcaacaagcgattgaagacaaaggtgaac
cccacaactcacatgtacacacattgtgagattcaccccagtatgattctcggtatctgt
gccagtatcattc
>SP_FOA05
catttgcgtcgaaccaatactcccatcggacgagatggtaaattggccaagcctcgacag
cttcacaacactcactggggtttggtgtgtcctgccgaaacacctgagggtcaagcttgt
ggtctggtcaaaaacttgtctctaatgtgttacgtcagtgtcggctctccagccgatcct
ctgattgaattcatgatcaacagaggcatggaagtcgttgaggagtacgagccgacaaga
tacccccacgctacaaagattttcgtcaacggtagctgggttggtgttcatgccgacccc
aagcatctcgtgaatcaggtcttggacacaagacgaaagtcttacgtgcagttcgaagta
tcacttgttcgtgatatccgagaccgtgaattcaagattttctcagacgctggccgtgtc
atgagacccgtctttacagttcatcaggaggatgactatgagaacaacatcaccaaggga
caactagtgttgacaaaggaccatgtcaataggctagcccaagaacaggcagagcctcct
gccaacccagcggacaagtttggatgggatggcttgatccgcgaaggagctgtcgagtat

ctcgatgctgaggaagaagagacagccatgatttgcatgacgccagaggatctcgaactt
taccgtgagcaaaagaatgatgaagctacactcacagaagaagagaaacgggccaagcaa
gaggcagagaagagagagaacaagaggaggaccgcaacaagcgattgaagacaaaggtgaac
cccacaactcacatgtacacacattgtgagattcaccccagtatgattctcggtatctgt
gccagtatcattc
>SP_FOA06
catttgcgtcgaaccaatactcccatcggacgagatggtaaattggccaagcctcgacag
cttcacaacactcactggggtttggtgtgtcctgccgaaacacctgagggtcaagcttgt
ggtctggtcaaaaacttgtctctaatgtgttacgtcagtgtcggctctccagccgatcct
ctgattgaattcatgatcaacagaggcatggaagtcgttgaggagtacgagccgacaaga
tacccccacgctacaaagattttcgtcaacggtagctgggttggtgttcatgccgacccc
aagcatctcgtgaatcaggtcttggacacaagacgaaagtcttacgtgcagttcgaagta
tcacttgttcgtgatatccgagaccgtgaattcaagattttctcagacgctggccgtgtc
atgagacccgtctttacagttcatcaggaggatgactatgagaacaacatcaccaaggga
caactagtgttgacaaaggaccatgtcaataggctagcccaagaacaggcagagcctcct
gccaacccagcggacaagtttggatgggatggcttgatccgcgaaggagctgtcgagtat
ctcgatgctgaggaagaagagacagccatgatttgcatgacgccagaggatctcgaactt
taccgtgagcaaaagaatgatgaagctacactcacagaagaagagaaacgggccaagcaa
gaggcagagaagagagagaacaagaggaggaccgcaacaagcgattgaagacaaaggtgaac
cccacaactcacatgtacacacattgtgagattcaccccagtatgattctcggtatctgt
gccagtatcattc
>SP_FOA07
catttgcgtcgaaccaatactcccatcggacgagatggtaaattggccaagcctcgacag
cttcacaacactcactggggtttggtgtgtcctgccgaaacacccgagggtcaagcttgt
ggtctggtcaaaaacttgtctctgatgtgttacgtcagtgttggctctccggccgatcct
ctgattgaattcatgatcaacagaggcatggaagtcgttgaggagtacgagccgacaaga
tacccccacgctacaaagattttcgtcaacggtagctgggttggtgttcatgccgacccc
aagcatctcgtgaatcaggtcttggacacaaggcgaaagtcttacgtgcagttcgaagta
tcacttgttcgtgatatccgagaccgtgaattcaagattttctcagacgctggccgtgtc
atgagacccgtctttacagttcatcaggaggatgactatgagaacaacatcaccaaggga
caactagtgttgacaaaggaccatgtcaataggctagcccaagagcaggcagagcctcct
gccaacccagcggacaagtttggatgggatggcttgatccgcgaaggagctgtcgagtat
ctcgatgctgaggaagaagagacagccatgatttgcatgacgccagaggatctcgaactt
taccgtgagcaaaagaatgatgaagctacactcacagaagaagagaaacgggccaagcaa
gaggcagagaagagggaacaagaggaggaccgcaacaagcgattgaagacaaaggtgaac
cccacaactcacatgtacacacattgtgagattcaccccagtatgattctcggtatctgt
gccagtatcattc
>SP_FOA08
catttgcgtcgaaccaatactcccatcggacgagatggtaaattggccaagcctcgacag
cttcacaacactcactggggtttggtgtgtcctgccgaaacacccgagggtcaagcttgt
ggtctggtcaaaaacttgtctctgatgtgttacgtcagtgttggctctccggccgatcct
ctgattgaattcatgatcaacagaggcatggaagtcgttgaggagtacgagccgacaaga
tacccccacgctacaaagattttcgtcaacggtagctgggttggtgttcatgccgacccc
aagcatctcgtgaatcaggtcttggacacaaggcgaaagtcttacgtgcagttcgaagta
tcacttgttcgtgatatccgagaccgtgaattcaagattttctcagacgctggccgtgtc
atgagacccgtctttacagttcatcaggaggatgactatgagaacaacatcaccaaggga
caactagtgttgacaaaggaccatgtcaataggctagcccaagagcaggcagagcctcct
gccaacccagcggacaagtttggatgggatggcttgatccgcgaaggagctgtcgagtat
ctcgatgctgaggaagaagagacagccatgatttgcatgacgccagaggatctcgaactt
taccgtgagcaaaagaatgatgaagctacactcacagaagaagagaaacgggccaagcaa
gaggcagagaagagggaacaagaggaggaccgcaacaagcgattgaagacaaaggtgaac
cccacaactcacatgtacacacattgtgagattcaccccagtatgattctcggtatctgt
gccagtatcattc
>SP_FOA09
catttgcgtcgaaccaatactcccatcggacgagatggtaaattggccaagcctcgacag
cttcacaacactcactggggtttggtgtgtcctgccgaaacacccgagggtcaagcttgt
ggtctggtcaaaaacttgtctctgatgtgttacgtcagtgttggctctccggccgatcct
ctgattgaattcatgatcaacagaggcatggaagtcgttgaggagtacgagccgacaaga
tacccccacgctacaaagattttcgtcaacggtagctgggttggtgttcatgccgacccc
aagcatctcgtgaatcaggtcttggacacaaggcgaaagtcttacgtgcagttcgaagta
tcacttgttcgtgatatccgagaccgtgaattcaagattttctcagacgctggccgtgtc

atgagacccgtctttacagttcatcaggaggatgactatgagaacaacatcaccaaggga
caactagtgttgacaaaggaccatgtcaataggctagcccaagagcaggcagagcctcct
gccaacccagcggacaagtttggatgggatggcttgatccgcgaaggagctgtcgagtat
ctcgatgctgaggaagaagagacagccatgatttgcatgacgccwgaggatctcgaactt
taccgtgagcaaaagaatgatgaagctacactcacagaagaagagaaacgggccaagcaa
gaggcagagaagagagagaacaagaggaggaccgcaacaagcgattgaagacaaaggtgaac
cccacaactcacatgtacacacattgtgagattcaccccagtatgattctcggtatctgt
gccagtatcattc
>SP_FOA10
catttgcgtcgaaccaatactcccatcggacgagatggtaaattggccaagcctcgacag
cttcacaacactcactggggtttggtgtgtcctgccgaaacacctgagggtcaagcttgt
ggtctggtcaaaaacttgtctctaatgtgttacgtcagtgtcggctctccagccgatcct
ctgattgaattcatgatcaacagaggcatggaagtcgttgaggagtacgagccgacaaga
taccccccacgctacaaagattttcgtcaacggtagctgggttggtgttcatgccgacccc
aagcatctcgtgaatcaggtcttggacacaagacgaaagtcttacgtgcagttcgaagta
tcacttgttcgtgatatccgagaccgtgaattcaagattttctcagacgctggccgtgtc
atgagacccgtctttacagttcatcaggaggatgactatgagaacaacatcaccaaggga
caactagtgttgacaaaggaccatgtcaataggctagcccaagaacaggcagagcctcct
gccaacccagcggacaagtttggatgggatggcttgatccgcgaaggagctgtcgagtat
ctcgatgctgaggaagaagagacagccatgatttgcatgacgccagaggatctcgaactt
taccgtgagcaaaagaatgatgaagctacactcacagaagaagagaaacgggccaagcaa
gaggcagagaagagagagaacaagaggaggaccgcaacaagcgattgaagacaaaggtgaac
cccacaactcacatgtacacacattgtgagattcaccccagtatgattctcggtatctgt
gccagtatcattc
>SP_FOA11
catttgcgtcgaaccaatactcccatcggacgagatggtaaattggccaagcctcgacag
cttcacaacactcactggggtttggtgtgtcctgccgaaacacccgagggtcaagcttgt
ggtctggtcaaaaacttgtctctgatgtgttacgtcagtgttggctctccggccgatcct
ctgattgaattcatgatcaacagaggcatggaagtcgttgaggagtacgagccgacaaga
taccccccacgctacaaagattttcgtcaacggtagctgggttggtgttcatgccgacccc
aagcatctcgtgaatcaggtcttggacacaaggcgaaagtcttacgtgcagttcgaagta
tcacttgttcgtgatatccgagaccgtgaattcaagattttctcagacgctggccgtgtc
atgagacccgtctttacagttcatcaggaggatgactatgagaacaacatcaccaaggga
caactagtgttgacaaaggaccatgtcaataggctagcccaagagcaggcagagcctcct
gccaacccagcggacaagtttggatgggatggcttgatccgcgaaggagctgtcgagtat
ctcgatgctgaggaagaagagacagccatgatttgcatgacgccagaggatctcgaactt
taccgtgagcaaaagaatgatgaagctacactcacagaagaagagaaacgggccaagcaa
gaggcagagaagagggaacaagaggaggaccgcaacaagcgattgaagacaaaggtgaac
cccacaactcacatgtacacacattgtgagattcaccccagtatgattctcggtatctgt
gccagtatcattc
>SP_FOA12
catttgcgtcgaaccaatactcccatcggacgagatggtaaattggccaagcctcgacag
cttcacaacactcactggggtttggtgtgtcctgccgaaacacccgagggtcaagcttgt
ggtctggtcaaaaacttgtctctgatgtgttacgtcagtgttggctctccggccgatcct
ctgattgaattcatgatcaacagaggcatggaagtcgttgaggagtacgagccgacaaga
taccccccacgctacaaagattttcgtcaacggtagctgggttggtgttcatgccgacccc
aagcatctcgtgaatcaggtcttggacacaaggcgaaagtcttacgtgcagttcgaagta
tcacttgttcgtgatatccgagaccgtgaattcaagattttctcagacgctggccgtgtc
atgagacccgtctttacagttcatcaggaggatgactatgagaacaacatcaccaaggga
caactagtgttgacaaaggaccatgtcaataggctagcccaagagcaggcagagcctcct
gccaacccagcggacaagtttggatgggatggcttgatccgcgaaggagctgtcgagtat
ctcgatgctgaggaagaagagacagccatgatttgcatgacgccagaggatctcgaactt
taccgtgagcaaaagaatgatgaagctacactcacagaagaagagaaacgggccaagcaa
gaggcagagaagagggaacaagaggaggaccgcaacaagcgattgaagacaaaggtgaac
cccacaactcacatgtacacacattgtgagattcaccccagtatgattctcggtatctgt
gccagtatcattc
>SP_FOA13
catttgcgtcgaaccaatactcccatcggacgagatggtaaattggccaagcctcgacag
cttcacaacactcactggggtttggtgtgtcctgccgaaacacctgagggtcaagcttgt
ggtctggtcaaaaacttgtctctaatgtgttacgtcagtgtcggctctccagccgatcct
ctgattgaattcatgatcaacagaggcatggaagtcgttgaggagtacgagccgacaaga

tacccccacgctacaaagattttcgtcaacggtagctgggttggtgttcatgccgacccc
aagcatctcgtgaatcaggtcttggacacaagacgaaagtcttacgtgcagttcgaagta
tcacttgttcgtgatatccgagaccgtgaattcaagattttctcagacgctggccgtgtc
atgagacccgtctttacagttcatcaggaggatgactatgagaacaacatcaccaaggga
caactagtgttgacaaaggaccatgtcaataggctagcccaagaacaggcagagcctcct
gccaacccagcggacaagtttggatgggatggcttgatccgcgaaggagctgtcgagtat
ctcgatgctgaggaagaagagacagccatgatttgcatgacgccagaggatctcgaactt
taccgtgagcaaaagaatgatgaagctacactcacagaagaagagaaacgggccaagcaa
gaggcagagaagagagagaacaagaggaggaccgcaacaagcgattgaagacaaaggtgaac
cccacaactcacatgtacacacattgtgagattcaccccagtatgattctcggtatctgt
gccagtatcattc
>SP_FOA14
catttgcgtcgaaccaatactcccatcggacgagatggtaaattggccaagcctcgacag
cttcacaacactcactggggtttggtgtgtcctgccgaaacacctgagggtcaagcttgt
ggtctggtcaaaaacttgtctctaatgtgttacgtcagtgtcggctctccagccgatcct
ctgattgaattcatgatcaacagaggcatggaagtcgttgaggagtacgagccgacaaga
tacccccacgctacaaagattttcgtcaacggtagctgggttggtgttcatgccgacccc
aagcatctcgtgaatcaggtcttggacacaagacgaaagtcttacgtgcagttcgaagta
tcacttgttcgtgatatccgagaccgtgaattcaagattttctcagacgctggccgtgtc
atgagacccgtctttacagttcatcaggaggatgactatgagaacaacatcaccaaggga
caactagtgttgacaaaggaccatgtcaataggctagcccaagaacaggcagagcctcct
gccaacccagcggacaagtttggatgggatggcttgatccgcgaaggagctgtcgagtat
ctcgatgctgaggaagaagagacagccatgatttgcatgacgccagaggatctcgaactt
taccgtgagcaaaagaatgatgaagctacactcacagaagaagagaaacgggccaagcaa
gaggcagagaagagagagaacaagaggaggaccgcaacaagcgattgaagacaaaggtgaac
cccacaactcacatgtacacacattgtgagattcaccccagtatgattctcggtatctgt
gccagtatcattc
>SP_FOA15
catttgcgtcgaaccaatactcccatcggacgagatggtaaattggccaagcctcgacag
cttcacaacactcactggggtttggtgtgtcctgccgaaacacctgagggtcaagcttgt
ggtctggtcaaaaacttgtctctaatgtgttacgtcagtgtcggctctccagccgatcct
ctgattgaattcatgatcaacagaggcatggaagtcgttgaggagtacgagccgacaaga
tacccccacgctacaaagattttcgtcaacggtagctgggttggtgttcatgccgacccc
aagcatctcgtgaatcaggtcttggacacaagacgaaagtcttacgtgcagttcgaagta
tcacttgttcgtgatatccgagaccgtgaattcaagattttctcagacgctggccgtgtc
atgcgacccgtctttacagttcatcaggaggatgactatgagaacaacatcaccaaggga
caactagtgttgacaaaggaccatgtcaataggctagcccaagaacaggcagagcctcct
gccaacccagcggacaagtttggatgggatggcttgatccgcgaaggagctgtcgagtat
ctcgatgctgaggaagaagagacagccatgatttgcatgacgccagaggatctcgaactt
taccgtgagcaaaagaatgatgaagctacactcacagaagaagagaaacgggccaagcaa
gaggcagagaagagagagaacaagaggaggaccgcaacaagcgattgaagacaaaggtgaac
cccacaactcacatgtacacacattgtgagattcaccctagtatgattctcggtatctgt
gccagtatcattc
>SP_FOA16
catttgcgtcgaaccaatactcccatcggacgagatggtaaattggccaagcctcgacag
cttcacaacactcactggggtttggtgtgtcctgccgaaacacctgagggtcaagcttgt
ggtctggtcaaaaacttgtctctaatgtgttacgtcagtgtcggctctccagccgatcct
ctgattgaattcatgatcaacagaggcatggaagtcgttgaggagtacgagccgacaaga
tacccccacgctacaaagattttcgtcaacggtagctgggttggtgttcatgccgacccc
aagcatctcgtgaatcaggtcttggacacaagacgaaagtcttacgtgcagttcgaagta
tcacttgttcgtgatatccgagaccgtgaattcaagattttctcagacgctggccgtgtc
atgcgacccgtctttacagttcatcaggaggatgactatgagaacaacatcaccaaggga
caactagtgttgacaaaggaccatgtcaataggctagcccaagaacaggcagagcctcct
gccaacccagcggacaagtttggatgggatggcttgatccgcgaaggagctgtcgagtat
ctcgatgctgaggaagaagagacagccatgatttgcatgacgccagaggatctcgaactt
taccgtgagcaaaagaatgatgaagctacactcacagaagaagagaaacgggccaagcaa
gaggcagagaagagagagaacaagaggaggaccgcaacaagcgattgaagacaaaggtgaac
cccacaactcacatgtacacacattgtgagattcaccctagtatgattctcggtatctgt
gccagtatcattc
>SP_FOA17
catttgcgtcgaaccaatactcccatcggacgagatggtaaattggccaagcctcgacag

cttcacaacactcactggggtttggtgtgtcctgccgaaacacctgagggtcaagcttgt
ggtctggtcaaaaacttgtctctaatgtgttacgtcagtgtcggctctccagccgatcct
ctgattgaattcatgatcaacagaggcatggaagtcgttgaggagtacgagccgacaaga
taccccccacgctacaaagattttcgtcaacggtagctgggttggtgttcatgccgacccc
aagcatctcgtgaatcaggtcttggacacaagacgaaagtcttacgtgcagttcgaagta
tcacttgttcgtgatatccgagaccgtgaattcaagattttctcagacgctggccgtgtc
atgcgacccgtctttacagttcatcaggaggatgactatgagaacaacatcaccaaggga
caactagtgttgacaaaggaccatgtcaataggctagcccaagaacaggcagagcctcct
gccaacccagcggacaagtttggatgggatggcttgatccgcgaaggagctgtcgagtat
ctcgatgctgaggaagaagagacagccatgatttgcatgacgccagaggatctcgaactt
taccgtgagcaaaagaatgatgaagctacactcacagaagaagagaaacgggccaagcaa
gaggcagagaagagagagaacaagaggaggaccgcaacaagcgattgaagacaaaggtgaac
cccacaactcacatgtacacacattgtgagattcaccctagtatgattctcggtatctgt
gccagtatcattc
>SP_FOA18
catttgcgtcgaaccaatactcccatcggacgagatggtaaattggccaagcctcgacag
cttcacaacactcactggggtttggtgtgtcctgccgaaacacctgagggtcaagcttgt
ggtctggtcaaaaacttgtctctaatgtgttacgtcagtgtcggctctccagccgatcct
ctgattgaattcatgatcaacagaggcatggaagtcgttgaggagtacgagccgacaaga
taccccccacgctacaaagattttcgtcaacggtagctgggttggtgttcatgccgacccc
aagcatctcgtgaatcaggtcttggacacaagacgaaagtcttacgtgcagttcgaagta
tcacttgttcgtgatatccgagaccgtgaattcaagattttctcagacgctggccgtgtc
atgcgacccgtctttacagttcatcaggaggatgactatgagaacaacatcaccaaggga
caactagtgttgacaaaggaccatgtcaataggctagcccaagaacaggcagagcctcct
gccaacccagcggacaagtttggatgggatggcttgatccgcgaaggagctgtcgagtat
ctcgatgctgaggaagaagagacagccatgatttgcatgacgccagaggatctcgaactt
taccgtgagcaaaagaatgatgaagctacactcacagaagaagagaaacgggccaagcaa
gaggcagagaagagagagaacaagaggaggaccgcaacaagcgattgaagacaaaggtgaac
cccacaactcacatgtacacacattgtgagattcaccctagtatgattctcggtatctgt
gccagtatcattc
>SP_FOA19
catttgcgtcgaaccaatactcccatcggacgagatggtaaattggccaagcctcgacag
cttcacaacactcactggggtttggtgtgtcctgccgaaacacctgagggtcaagcttgt
ggtctggtcaaaaacttgtctctaatgtgttacgtcagtgtcggctctccagccgatcct
ctgattgaattcatgatcaacagaggcatggaagtcgttgaggagtacgagccgacaaga
taccccccacgctacaaagattttcgtcaacggtagctgggttggtgttcatgccgacccc
aagcatctcgtgaatcaggtcttggacacaagacgaaagtcttacgtgcagttcgaagta
tcacttgttcgtgatatccgagaccgtgaattcaagattttctcagacgctggccgtgtc
atgcgacccgtctttacagttcatcaggaggatgactatgagaacaacatcaccaaggga
caactagtgttgacaaaggaccatgtcaataggctagcccaagaacaggcagagcctcct
gccaacccagcggacaagtttggatgggatggcttgatccgcgaaggagctgtcgagtat
ctcgatgctgaggaagaagagacagccatgatttgcatgacgccagaggatctcgaactt
taccgtgagcaaaagaatgatgaagctacactcacagaagaagagaaacgggccaagcaa
gaggcagagaagagagagaacaagaggaggaccgcaacaagcgattgaagacaaaggtgaac
cccacaactcacatgtacacacattgtgagattcaccctagtatgattctcggtatctgt
gccagtatcattc
>SP_FOA20
catttgcgtcgaaccaatactcccatcggacgagatggtaaattggccaagcctcgacag
cttcacaacactcactggggtttggtgtgtcctgccgaaacacctgagggtcaagcttgt
ggtctggtcaaaaacttgtctctaatgtgttacgtcagtgtcggctctccagccgatcct
ctgattgaattcatgatcaacagaggcatggaagtcgttgaggagtacgagccgacaaga
taccccccacgctacaaagattttcgtcaacggtagctgggttggtgttcatgccgacccc
aagcatctcgtgaatcaggtcttggacacaagacgaaagtcttacgtgcagttcgaagta
tcacttgttcgtgatatccgagaccgtgaattcaagattttctcagacgctggccgtgtc
atgcgacccgtctttacagttcatcaggaggatgactatgagaacaacatcaccaaggga
caactagtgttgacaaaggaccatgtcaataggctagcccaagaacaggcagagcctcct
gccaacccagcggacaagtttggatgggatggcttgatccgcgaaggagctgtcgagtat
ctcgatgctgaggaagaagagacagccatgatttgcatgacgccagaggatctcgaactt
taccgtgagcaaaagaatgatgaagctacactcacagaagaagagaaacgggccaagcaa
gaggcagagaagagagagaacaagaggaggaccgcaacaagcgattgaagacaaaggtgaac
cccacaactcacatgtacacacattgtgagattcaccctagtatgattctcggtatctgt

gccagtatcattc

S3: Final alignments in FASTA format (RPB2);

```
>fusarium_proliferatum_et1_gca_900067095
gagatgaaccttcacgtcccacagagtgaggaaacacgtgctgaggttaaagagctttgt
ctagtccccctgaacattgtctctcctcagaagaacggtcctttgatgggtatcgtccag
gactctctggctggtgcctacaagctttgccgtcgagatgttttcctcacaaaggagcag
atcatgaactgtatgctctgggtgcctaattgggacggtgtcattcctcagcccgctatc
tataagcctcgtcctcggtggactggtaagcagcttatcagcatggtcatccctaaggag
gtaagcctgttcaatggtacggattctggtgaaaacgcccctcttaaggacgagggtctt
ctgatccaagccggccaactgatgtatggtcttttgactaagaagaacattggtgctgct
gcgggcggtattgtgcatatcagctacaacgaacttggccccgaaggtgcgatggctttc
ctgaacggtgtccagcaggttgtcacctactggcttctcaacaatggtcatagcattggt
attggtgatacaattcccgatgcggcgaccattgctaaagtccaggtgcacattgacgag
gaaaaggctgaagttgctcgcttgacagcaatggccacagcgaacgagcttgaggctcta
ccaggtatgaacgttcgtgcaaccttcgaaaacaaggtctccatggctctgaatcaggcc
cgtgataaggctggtaccacaacacagaagagtttgaaggattcaaacaacgctgtcacc
atggcttcctcaggttccaagggttcatctatcaatatttctcaaatgactgcgcttgtc
ggtcagcaaattgttgaaggcaagcgtattcctttcggtttcaagtatcgcacattacct
cacttcaccaaggac
>fusarium_oxysporum_fo47_gca_000271705
gagatgaaccttcacgtcccccagagtgaggaaacgcgcgctgaggttaaagagctttgt
ctagttcccttgaacattgtctctcctcagaagaacggtcctttgatgggtatcgtccag
gactctctggctggtgcctacaagctttgccgtcgagatgttttcctcacaaaggagcaa
atcatgaactgtatgctctgggtgcccaactgggacggtgtcattcctcaacccgctatc
tataagcctcgtcctcggtggactggtaagcagcttatcagcatggttatccctaaggag
gttagcctgttcaacggtacggattctggcgaaaacgcccctcttaaggacgagggtctt
ctgatccaagccggccaactgatgtatggtcttttaactaagaagaacattggtgctgct
gcgggtggtattgtgcatatcagctacaacgaacttggccccgaaggtgcgatggctttc
ttaaacggtgtccagcaggttgtcacctactggcttctcaacaatggtcatagcattggt
attggtgatacaattcccgatgcggcgaccattgctaaagttcaggtacatattgatgag
gaaaaggctgaagttgctcgcttgacagcaatggccacagcgaatgagcttgaggcccta
cctggtatgaacgttcgtgcaaccttcgaaaacaaagtctccatggctctgaaccaggcc
cgtgataaggctggtaccacaacacagaagagtttgaaggattcaaacaacgctgtcacc
atggcttcctcaggttccaagggttcatctatcaatatttctcaaatgactgcgcttgtc
ggtcagcaaattgtcgaaggcaagcgtattccttttggtttcaagtatcgcacattacct
cacttcaccaaggac
>GASP_2 no comment
gagatgaaccttcacgtcccccagagtgaggaaacgcgcgctgaggttaaagagctttgt
ctagttcccttgaacattgtctctcctcagaagaacggtcctttgatgggtatcgtccag
gactctctggctggtgcctacaagctttgccgtcgagatgttttcctcacaaaggagcaa
atcatgaactgtatgctctgggtgcccaactgggacggtgtcattcctcaacccgctatc
tataagcctcgtcctcggtggactggtaagcagcttatcagcatggttatccctaaggag
gttagcctgttcaacggtacggattctggcgaaaacgcccctcttaaggacgagggtctt
ctgatccaagccggccaactgatgtatggtcttttaactaagaagaacattggtgctgct
gcgggtggtattgtgcatatcagctacaacgaacttggccccgaaggtgcgatggctttc
ttaaacggtgtccagcaggttgtcacctactggcttctcaacaatggtcatagcattggt
attggtgatacaattcccgatgcggcgaccattgctaaagttcaggtacatattgatgag
gaaaaggctgaagttgctcgcttgacagcaatggccacagcgaatgagcttgaggcccta
cctggtatgaacgttcgtgcaaccttcgaaaacaaagtctccatggctctgaaccaggcc
cgtgataaggctggtaccacaacacagaagagtttgaaggattcaaacaacgctgtcacc
atggcttcctcaggttccaagggttcatctatcaatatttctcaaatgactgcgcttgtc
ggtcagcaaattgtcgaaggcaagcgtattccttttggtttcaagtatcgcacattacct
cacttcaccaaggac
>GASP_3 no comment
gagatgaaccttcacgtcccccagagtgaggaaacgcgcgctgaggttaaagagctttgt
ctagttcccttgaacattgtctctcctcagaagaacggtcctttgatgggtatcgtccag
gactctctggctggtgcctacaagctttgccgtcgagatgttttcctcacaaaggagcaa
```

atcatgaactgtatgctctgggtgcccaactgggacggtgtcattcctcaacccgctatc
tataagcctcgtcctcggtggactggtaagcagcttatcagcatggttatccctaaggag
gttagcctgttcaacggtacggattctggcgaaaacgcccctcttaaggacgagggtctt
ctgatccaagccggccaactgatgtatggtcttttaactaagaagaacattggtgctgct
gcgggtggtattgtgcatatcagctacaacgaacttggccccgaaggtgcgatggctttc
ttaaacggtgtccagcaggttgtcacctactggcttctcaacaatggtcatagcattggt
attggtgatacaattcccgatgcggcgaccattgctaaagttcaggtacatattgatgag
gaaaaggctgaagttgctcgcttgacagcaatggccacagcgaatgagcttgaggcccta
cctggtatgaacgttcgtgcaaccttcgaaaacaaagtctccatggctctgaaccaggcc
cgtgataaggctggtaccacaacacagaagagtttgaaggattcaaacaacgctgtcacc
atggcttcctcaggttccaagggttcatctatcaatatttctcaaatgactgcgcttgtc
ggtcagcaaattgtcgaaggcaagcgtattccttttggtttcaagtatcgcacattacct
cacttcaccaaggac
>GASP_4 no comment
gagatgaaccttcacgtcccccagagcgaggaaacgcgcgctgaggttaaagagctttgt
ctagttcccttgaacattgtctctcctcagaagaacggtcctttgatgggtatcgttcag
gactctctggctggtgcctacaagctttgccgtcgagatgttttcctcacaaaggagcaa
atcatgaactgtatgctctgggtgcccaactgggacggtgtcattcctcaacccgctatc
tataagcctcgtcctcggtggactggtaagcagcttatcagcatggttatccctaaggag
gttagcctgttcaacggtacggattctggtgaaaacgcccctcttaaggacgagggtctt
ctgatccaagccggccaactgatgtatggtcttttaactaagaagaacattggtgctgct
gcgggtggtattgtgcatatcagctacaacgaacttggccccgaaggtgcgatggctttc
ttaaacggtgtccagcaggttgtcacctactggcttctcaacaatggtcatagcattggt
attggtgatacaattcccgatgcggcgaccattgctaaagttcaggtacatattgatgag
gaaaaggctgaagttgctcgcttgacagcaatggccacagcgaatgagcttgaggcccta
cctggt--

>GASP_5 no comment
gagatgaaccttcacgtccctcagagtgaggaaacgcgcgctgaggttaaagagctttgt
ctagttcccttgaacattgtctctcctcagaagaacggtcctttgatgggtatcgtccag
gactctctggctggtgcctacaagctttgccgtcgagatgttttcctcacaaaggagcaa
atcatgaactgtatgctctgggtgcccaactgggacggtgtcattcctcaacccgctatc
tataagcctcgtcctcggtggactggtaagcagcttatcagcatggttatccctaaggag
gttagcctgttcaacggtacggattctggcgaaaacgcccctcttaaggacgagggtctt
ctgatccaagccggccaactgatgtatggtcttttaactaagaagaacattggtgctgct
gcgggtggtattgtgcatatcagctacaacgaacttggccccgaaggtgcgatggctttc
ttaaacggtgtccagcaggttgtcacctactggcttctcaacaatggtcatagcattggt
attggtgatacaattcccgatgcggcgaccattgctaaagttcaggtacatattgatgag
gaaaaggctgaagttgctcgcttgacagcaatggccacagcgaatgagcttgaggcccta
cctggtatgaacgttcgtgcaaccttcgaaaacaaagtctccatggctctgaaccaggcc
cgtgataaggctggtaccacaacacagaagagtttgaaggattcaaacaacgctgtcacc
atggcttcctcaggttccaagggttcatctatcaatatttctcaaatgactgcgcttgtc
ggtcagcaaattgttgaaggcaagcgtattccttttggtttcaagtatcgcacattacct
cacttcaccaaggac
>GASP_9 no comment
gagatgaaccttcacgtcccccagagtgaggaaacgcgcgctgaggttaaagagctttgt
ctagttcccttgaacattgtctctcctcagaagaacggtcctttgatgggtatcgtccag
gactctctggctggtgcctacaagctttgccgtcgagatgttttcctcacaaaggagcaa
atcatgaactgtatgctctgggtgcccaactgggacggtgtcattcctcaacccgctatc
tataagcctcgtcctcggtggactggtaagcagcttatcagcatggttatccctaaggag
gttagcctgttcaacggtacggattctggcgaaaacgcccctcttaaggacgagggtctt
ctgatccaagccggccaactgatgtatggtcttttaactaagaagaacattggtgctgct
gcgggtggtattgtgcatatcagctacaacgaacttggccccgaaggtgcgatggctttc
ttaaacggtgtccagcaggttgtcacctactggcttctcaacaatggtcatagcattggt
attggtgatacaattcccgatgcggcgaccattgctaaagttcaggtacatattgatgag
gaaaaggctgaagttgctcgcttgacagcaatggccacagcgaatgagcttgaggcccta
cctggtatgaacgttcgtgcaaccttcgaaaacaaagtctccatggctctgaaccaggcc
cgtgataaggctggtaccacaacacagaagagtttgaaggattcaaacaacgctgtcacc

atggcttcctcaggttccaagggttcatctatcaatatttctcaaatgactgcgcttgtc
ggtcagcaaattgtcgaaggcaagcgtattccttttggtttcaagtatcgcacattacct
cacttcaccaaggac
>GA_1.2 no comment
gagatgaaccttcacgtcccccagagtgaggaaacgcgcgctgaggttaaagagctttgt
ctagttcccttgaacattgtctctcctcagaagaacggtcctttgatgggtatcgtccag
gactctctggctggtgcctacaagctttgccgtcgagatgttttcctcacaaaggagcaa
atcatgaactgtatgctctgggtgcccaactgggacggtgtcattcctcaacccgctatc
tataagcctcgtcctcggtggactggtaagcagcttatcagcatggttatccctaaggag
gttagcctgttcaacggtacggattctggcgaaaacgcccctcttaaggacgagggtctt
ctgatccaagccggccaactgatgtatggtcttttaactaagaagaacattggtgctgct
gcgggtggtattgtgcatatcagctacaacgaacttggccccgaaggtgcgatggctttc
ttaaacggtgtccagcaggttgtcacctactggcttctcaacaatggtcatagcattggt
attggtgatacaattcccgatgcggcgaccattgctaaagttcaggtacatattgatgag
gaaaaggctgaagttgctcgcttgacagcaatggccacagcgaatgagcttgaggcccta
cctggtatgaacgttcgtgcaaccttcgaaaacaaagtctccatggctctgaaccaggcc
cgtgataaggctggtaccacaacacagaagagtttgaaggattcaaacaacgctgtcacc
atggcttcctcaggttccaagggttcatctatcaatatttctcaaatgactgcgcttgtc
ggtcagcaaattgtcgaaggcaagcgtattccttttggtttcaagtatcgcacattacct
cacttcaccaaggac
>GA_1.3 no comment
---tttgt
ctagttcccttgaacattgtctctcctcagaagaacggtcctttgatgggtatcgtccag
gactctctggctggtgcctacaagctttgccgtcgagatgttttcctcacaaaggagcaa
atcatgaactgtatgctctgggtgcccaactgggacggtgtcattcctcaacccgctatc
tataagcctcgtcctcggtggactggtaagcagcttatcagcatggttatccctaaggag
gttagcctgttcaacggtacggattctggcgaaaacgcccctcttaaggacgagggtctt
ctgatccaagccggccaactgatgtatggtcttttaactaagaagaacattggtgctgct
gcgggtggtattgtgcatatcagctacaacgaacttggccccgaaggtgcgatggctttc
ttaaacggtgtccagcaggttgtcacctactggcttctcaacaatggtcatagcattggt
attggtgatacaattcccgatgcggcgaccattgctaaagttcaggtacatattgatgag
gaaaaggctgaagttgctcgcttgacagcaatggccacagcgaatgagcttgaggcccta
cctggtatgaacgttcgtgcaaccttcgaaaacaaagtctccatggctctgaaccaggcc
cgtgataaggctggtaccacaacacagaagagtttgaaggattcaaacaacgctgtcacc
atggcttcctcaggttccaagggttcatctatcaatatttctcaaatgactgcgcttgtc
ggtcagcaaattgtcgaaggcaagcgtattccttttggtttcaagtatcgcacattacct
cacttcaccaaggac
>GA_1.4 no comment
gagatgaaccttcacgtcccccagagtgaggaaacgcgcgctgaggttaaagagctttgt
ctagttcccttgaacattgtctctcctcagaagaacggtcctttgatgggtatcgtccag
gactctctggctggtgcctacaagctttgccgtcgagatgttttcctcacaaaggagcaa
atcatgaactgtatgctctgggtgcccaactgggacggtgtcattcctcaacccgctatc
tataagcctcgtcctcggtggactggtaagcagcttatcagcatggttatccctaaggag
gttagcctgttcaacggtacggattctggcgaaaacgcccctcttaaggacgagggtctt
ctgatccaagccggccaactgatgtatggtcttttaactaagaagaacattggtgctgct
gcgggtggtattgtgcatatcagctacaacgaacttggccccgaaggtgcgatggctttc
ttaaacggtgtccagcaggttgtcacctactggcttctcaacaatggtcatagcattggt
attggtgatacaattcccgatgcggcgaccattgctaaagttcaggtacatattgatgag
gaaaaggctgaagttgctcgcttgacagcaatggccacagcgaatgagcttgaggcccta
cctggtatgaacgttcgtgcaaccttcgaaaacaaagtctccatggctctgaaccaggcc
cgtgataaggctggtaccacaacacagaagagtttgaaggattcaaacaacgctgtcacc
atggcttcctcaggttccaagggttcatctatcaatatttctcaaatgactgcgcttgtc
ggtcagcaaattgtcgaaggcaagcgtattccttttggtttcaagtatcgcacattacct
cacttcaccaaggac
>GA_10 no comment
gagatgaaccttcacgtcccccagagtgaggaaacgcgcgctgaggttaaagagctttgt
ctagttcccttgaacattgtctctcctcagaagaacggtcctttgatgggtatcgtccag
gactctctggctggtgcctacaagctttgccgtcgagatgttttcctcacaaaggagcaa
atcatgaactgtatgctctgggtgcccaactgggacggtgtcattcctcaacccgctatc
tataagcctcgtcctcggtggactggtaagcagcttatcagcatggttatccctaaggag
gttagcctgttcaacggtacggattctggcgaaaacgcccctcttaaggacgagggtctt

ctgatccaagccggccaactgatgtatggtcttttaactaagaagaacattggtgctgct
gcgggtggtattgtgcatatcagctacaacgaacttggccccgaaggtgcgatggctttc
ttaaacggtgtccagcaggttgtcacctactggcttctcaacaatggtcatagcattggt
attggtgatacaattcccgatgcggcgaccattgctaaagttcaggtacatattgatgag
gaaaaggctgaagttgctcgcttgacagcaatggccacagcgaatgagcttgaggcccta
cctggtatgaacgttcgtgcaaccttcgaaaacaaagtctccatggctctgaaccaggcc
cgtgataaggctggtaccacaacacagaagagtttgaaggattcaaacaacgctgtcacc
atggcttcctcangttccaagggttcatctatcaatatttctcaaatgactgcgcttgtc
ggtcagcaaattgtcgaaggcaagcgtattccttttggtttcaagtatcgcacattacct
cacttnaccaaggac
>GA_3.2 no comment
gagatgaaccttcacgtcccccagagtgaggaaacgcgcgctgaggttaaagagctttgt
ctagttcccttgaacattgtctctcctcagaagaacggtcctttgatgggtatcgtccag
gactctctggctggtgcctacaagctttgccgtcgagatgttttcctcacaaaggagcaa
atcatgaactgtatgctctgggtgcccaactgggacggtgtcattcctcaacccgctatc
tataagcctcgtcctcggtggactggtaagcagcttatcagcatggttatccctaaggag
gttagcctgttcaacggtacggattctggcgaaaacgcccctcttaaggacgagggtctt
ctgatccaagccggccaactgatgtatggtcttttaactaagaagaacattggtgctgct
gcgggtggtattgtgcatatcagctacaacgaacttggccccgaaggtgcgatggctttc
ttaaacggtgtccagcaggttgtcacctactggcttctcaacaatggtcatagcattggt
attggtgatacaattcccgatgcggcgaccattgctaaagttcaggtacatattgatgag
gaaaaggctgaagttgctcgcttgacagcaatggccacagcgaatgagcttgaggcccta
cctggtatgaacgttcgtgcaaccttcgaaaacaaagtctccatggctctgaaccaggcc
cgtgataaggctggtaccacaacacagaagagtttgaaggattcaaacaacgctgtcacc
atggcttcctcaggttccaagggttcatctatcaatatttctcaaatgactgcgcttgtc
ggtcagcaaattgtcgaaggcaagcgtattccttttggtttcaagtatcgcacattacct
cacttcaccaaggac
>GA_3.3 no comment
gagatgaaccttcacgtcccccagagtgaggaaacgcgcgctgaggttaaagagctttgt
ctagttcccttgaacattgtctctcctcagaagaacggtcctttgatgggtatcgtccag
gactctctggctggtgcctacaagctttgccgtcgagatgttttcctcacaaaggagcaa
atcatgaactgtatgctctgggtgcccaactgggacggtgtcattcctcaacccgctatc
tataagcctcgtcctcggtggactggtaagcagcttatcagcatggttatccctaaggag
gttagcctgttcaacggtacggattctggcgaaaacgcccctcttaaggacgagggtctt
ctgatccaagccggccaactgatgtatggtcttttaactaagaagaacattggtgctgct
gcgggtggtattgtgcatatcagctacaacgaacttggccccgaaggtgcgatggctttc
ttaaacggtgtccagcaggttgtcacctactggcttctcaacaatggtcatagcattggt
attggtgatacaattcccgatgcggcgaccattgctaaagttcaggtacatattgatgag
gaaaaggctgaagttgctcgcttgacagcaatggccacagcgaatgagcttgaggcccta
cctggtatgaacgttcgtgcaaccttcgaaaacaaagtctccatggctctgaaccaggcc
cgtgataaggctggtaccacaacacagaagagtttgaaggattcaaacaacgctgtcacc
atggcttcctcaggttccaagggttcatctatcaatatttctcaaatgactgcgcttgtc
ggtcagcaaattgtcgaaggcaagcgtattccttttggtttcaagtatcgcacattacct
cacttcaccaaggac
>GA_3.5 no comment
gagatgaaccttcacgtcccccagagtgaggaaacgcgcgctgaggttaaagagctttgt
ctagttcccttgaacattgtctctcctcagaagaacggtcctttgatgggtatcgtccag
gactctctggctggtgcctacaagctttgccgtcgagatgttttcctcacaaaggagcaa
atcatgaactgtatgctctgggtgcccaactgggacggtgtcattcctcaacccgctatc
tataagcctcgtcctcggtggactggtaagcagcttatcagcatggttatccctaaggag
gttagcctgttcaacggtacggattctggcgaaaacgcccctcttaaggacgagggtctt
ctgatccaagccggccaactgatgtatggtcttttaactaagaagaacattggtgctgct
gcgggtggtattgtgcatatcagctacaacgaacttggccccgaaggtgcgatggctttc
ttaaacggtgtccagcaggttgtcacctactggcttctcaacaatggtcatagcattggt
attggtgatacaattcccgatgcggcgaccattgctaaagttcaggtacatattgatgag
gaaaaggctgaagttgctcgcttgacagcaatggccacagcgaatgagcttgaggcccta
cctggtatgaacgttcgtgcaaccttcgaaaacaaagtctccatggctctgaaccaggcc
cgtgataaggctggtaccacaacacagaagagtttgaaggattcaaacaacgctgtcacc
atggcttcctcaggttccaagggttcatctatcaatatttctcaaatgactgcgcttgtc
ggtcagcaaattgtcgaaggcaagcgtattccttttggtttcaagtatcgcacattacct
cacttcaccaaggac

>GA_3.6 no comment
gagatgaaccttcacgtcccccagagtgaggaaacgcgcgctgaggttaaagagctttgt
ctagttcccttgaacattgtctctcctcagaagaacggtcctttgatgggtatcgtccag
gactctctggctggtgcctacaagctttgccgtcgagatgttttcctcacaaaggagcaa
atcatgaactgtatgctctgggtgcccaactgggacggtgtcattcctcaacccgctatc
tataagcctcgtcctcggtggactggtaagcagcttatcagcatggttatccctaaggag
gttagcctgttcaacggtacggattctggcgaaaacgcccctcttaaggacgagggtctt
ctgatccaagccggccaactgatgtatggtcttttaactaagaagaacattggtgctgct
gcgggtggtattgtgcatatcagctacaacgaacttggccccgaaggtgcgatggctttc
ttaaacggtgtccagcaggttgtcacctactggcttctcaacaatggtcatagcattggt
attggtgatacaattcccgatgcggcgaccattgctaaagttcaggtacatattgatgag
gaaaaggctgaagttgctcgcttgacagcaatggccacagcgaatgagcttgaggcccta
cctggtatgaacgttcgtgcaaccttcgaaaacaaagtctccatggctctgaaccaggcc
cgtgataaggctggtaccacaacacagaagagtttgaaggattcaaacaacgctgtcacc
atggcttcctcaggttccaagggttcatctatcaatatttctcaaatgactgcgcttgtc
ggtcagcaaattgtcgaaggcaagcgtattccttttggtttcaagtatcgcacattacct
cacttcaccaaggac
>GA_3.7 no comment
gagatgaaccttcacgtcccccagagtgaggaaacgcgcgctgaggttaaagagctttgt
ctagttcccttgaacattgtctctcctcagaagaacggtcctttgatgggtatcgtccag
gactctctggctggtgcctacaagctttgccgtcgagatgttttcctcacaaaggagcaa
atcatgaactgtatgctctgggtgcccaactgggacggtgtcattcctcaacccgctatc
tataagcctcgtcctcggtggactggtaagcagcttatcagcatggttatccctaaggag
gttagcctgttcaacggtacggattctggcgaaaacgcccctcttaaggacgagggtctt
ctgatccaagccggccaactgatgtatggtcttttaactaagaagaacattggtgctgct
gcgggtggtattgtgcatatcagctacaacgaacttggccccgaaggtgcgatggctttc
ttaaacggtgtccagcaggttgtcacctactggcttctcaacaatggtcatagcattggt
attggtgatacaattcccgatgcggcgaccattgctaaagttcaggtacatattgatgag
gaaaaggctgaagttgctcgcttgacagcaatggccacagcgaatgagcttgaggcccta
cctggtatgaacgttcgtgcaaccttcgaaaacaaagtctccatggctctgaaccaggcc
cgtgataaggctggtaccacaacacagaagagtttgaaggattcaaacaacgctgtcacc
atggcttcctcaggttccaagggttcatctatcaatatttctcaaatgactgcgcttgtc
ggtcagcaaattgtcgaaggcaagcgtattccttttggtttcaagtatcgcacattacct
cacttcaccaaggac
>GA_4.1 no comment
gagatgaaccttcacgtcccccagagtgaggaaacgcgcgctgaggttaaagagctttgt
ctagttcccttgaacattgtctctcctcagaagaacggtcctttgatgggtatcgtccag
gactctctggctggtgcctacaagctttgccgtcgagatgttttcctcacaaaggagcaa
atcatgaactgtatgctctgggtgcccaactgggacggtgtcattcctcaacccgctatc
tataagcctcgtcctcggtggactggtaagcagcttatcagcatggttatccctaaggag
gttagcctgttcaacggtacggattctggcgaaaacgcccctcttaaggacgagggtctt
ctgatccaagccggccaactgatgtatggtcttttaactaagaagaacattggtgctgct
gcgggtggtattgtgcatatcagctacaacgaacttggccccgaaggtgcgatggctttc
ttaaacggtgtccagcaggttgtcacctactggcttctcaacaatggtcatagcattggt
attggtgatacaattcccgatgcggcgaccattgctaaagttcaggtacatattgatgag
gaaaaggctgaagttgctcgcttgacagcaatggccacagcgaatgagcttgaggcccta
cctggtatgaacgttcgtgcaaccttcgaaaacaaagtctccatggctctgaaccaggcc
cgtgataaggctggtaccacaacacagaagagtttgaaggattcaaacaacgctgtcacc
atggcttcctcaggttccaagggttcatctatcaatatttctcaaatgactgcgcttgtc
ggtcagcaaattgtcgaaggcaagcgtattccttttggtttcaagtatcgcacattacct
cacttcaccaaggac
>GA_4.2 no comment
gagatgaaccttcacgtcccccagagtgaggaaacgcgcgctgaggttaaagagctttgt
ctagttcccttgaacattgtctctcctcagaagaacggtcctttgatgggtatcgtccag
gactctctggctggtgcctacaagctttgccgtcgagatgttttcctcacaaaggagcaa
atcatgaactgtatgctctgggtgcccaactgggacggtgtcattcctcaacccgctatc
tataagcctcgtcctcggtggactggtaagcagcttatcagcatggttatccctaaggag
gttagcctgttcaacggtacggattctggcgaaaacgcccctcttaaggacgagggtctt
ctgatccaagccggccaactgatgtatggtcttttaactaagaagaacattggtgctgct
gcgggtggtattgtgcatatcagctacaacgaacttggccccgaaggtgcgatggctttc
ttaaacggtgtccagcaggttgtcacctactggcttctcaacaatggtcatagcattggt

attggtgatacaattcccgatgcggcgaccattgctaaagttcaggtacatattgatgag
gaaaaggctgaagttgctcgcttgacagcaatggccacagcgaatgagcttgaggcccta
cctggtatgaacgttcgtgcaaccttcgaaaacaaagtctccatggctctgaaccaggcc
cgtgataaggctggtaccacaacacagaagagtttgaaggattcaaacaacgctgtcacc
atggcttcctcaggttccaagggttcatctatcaatatttctcaaatgactgcgcttgtc
ggtcagcaaattgtcgaaggcaagcgtattccttttggtttcaagtatcgcacattacct
cacttcaccaaggac
>GA_4.3 no comment
gagatgaaccttcacgtcccccagagtgaggaaacgcgcgctgaggttaaagagctttgt
ctagttcccttgaacattgtctctcctcagaagaacggtcctttgatgggtatcgtccag
gactctctggctggtgcctacaagctttgccgtcgagatgttttcctcacaaaggagcaa
atcatgaactgtatgctctgggtgcccaactgggacggtgtcattcctcaacccgctatc
tataagcctcgtcctcggtggactggtaagcagcttatcagcatggttatccctaaggag
gttagcctgttcaacggtacggattctggcgaaaacgcccctcttaaggacgagggtctt
ctgatccaagccggccaactgatgtatggtcttttaactaagaagaacattggtgctgct
gcgggtggtattgtgcatatcagctacaacgaacttggccccgaaggtgcgatggctttc
ttaaacggtgtccagcaggttgtcacctactggcttctcaacaatggtcatagcattggt
attggtgatacaattcccgatgcggcgaccattgctaaagttcaggtacatattgatgag
gaaaaggctgaagttgctcgcttgacagcaatggccacagcgaatgagcttgaggcccta
cctggtatgaacgttcgtgcaaccttcgaaaacaaagtctccatggctctgaaccaggcc
cgtgataaggctggtaccacaacacagaagagtttgaaggattcaaacaacgctgtcacc
atggcttcctcaggttccaagggttcatctatcaatatttctcaaatgactgcgcttgtc
ggtcagcaaattgtcgaaggcaagcgtattccttttggtttcaagtatcgcacattacct
cacttcaccaaggac
>GA_5.3 no comment
gagatgaaccttcacgtcccccagagtgaggaaacgcgcgctgaggttaaagagctttgt
ctagttcccttgaacattgtctctcctcagaagaacggtcctttgatgggtatcgtccag
gactctctggctggtgcctacaagctttgccgtcgagatgttttcctcacaaaggagcaa
atcatgaactgtatgctctgggtgcccaactgggacggtgtcattcctcaacccgctatc
tataagcctcgtcctcggtggactggtaagcagcttatcagcatggttatccctaaggag
gttagcctgttcaacggtacggattctggcgaaaacgcccctcttaaggacgagggtctt
ctgatccaagccggccaactgatgtatggtcttttaactaagaagaacattggtgctgct
gcgggtggtattgtgcatatcagctacaacgaacttggccccgaaggtgcgatggctttc
ttaaacggtgtccagcaggttgtcacctactggcttctcaacaatggtcatagcattggt
attggtgatacaattcccgatgcggcgaccattgctaaagttcaggtacatattgatgag
gaaaaggctgaagttgctcgcttgacagcaatggccacagcgaatgagcttgaggcccta
cctggtatgaacgttcgtgcaaccttcgaaaacaaagtctccatggctctgaaccaggcc
cgtgataaggctggtaccacaacacagaagagtttgaaggattcaaacaacgctgtcacc
atggcttcctcaggttccaagggttcatctatcaatatttctcaaatgactgcgcttgtc
ggtcagcaaattgtcgaaggcaagcgtattccttttggtttcaagtatcgcacattacct
cacttcaccaaggac
>GA_5.4 no comment
gagatgaaccttcacgtcccccagagtgaggaaacgcgcgctgaggttaaagagctttgt
ctagttcccttgaacattgtctctcctcagaagaacggtcctttgatgggtatcgtccag
gactctctggctggtgcctacaagctttgccgtcgagatgttttcctcacaaaggagcaa
atcatgaactgtatgctctgggtgcccaactgggacggtgtcattcctcaacccgctatc
tataagcctcgtcctcggtggactggtaagcagcttatcagcatggttatccctaaggag
gttagcctgttcaacggtacggattctggcgaaaacgcccctcttaaggacgagggtctt
ctgatccaagccggccaactgatgtatggtcttttaactaagaagaacattggtgctgct
gcgggtggtattgtgcatatcagctacaacgaacttggccccgaaggtgcgatggctttc
ttaaacggtgtccagcaggttgtcacctactggcttctcaacaatggtcatagcattggt
attggtgatacaattcccgatgcggcgaccattgctaaagttcaggtacatattgatgag
gaaaaggctgaagttgctcgcttgacagcaatggccacagcgaatgagcttgaggcccta
cctggtatgaacgttcgtgcaaccttcgaaaacaaagtctccatggctctgaaccaggcc
cgtgataaggctggtaccacaacacagaagagtttgaaggattcaaacaacgctgtcacc
atggcttcctcaggttccaagggttcatctatcaatatttctcaaatgactgcgcttgtc
ggtcagcaaattgtcgaaggcaagcgtattccttttggtttcaagtatcgcacattacct
cacttcaccaaggac
>GA_92.3 no comment
gagatgaaccttcacgtcccccagagtgaggaaacgcgcgctgaggttaaagagctttgt
ctagttcccttgaacattgtctctcctcagaagaacggtcctttgatgggtatcgtccag

gactctctggctggtgcctacaagctttgccgtcgagatgttttcctcacaaaggagcaa
atcatgaactgtatgctctgggtgcccaactgggacggtgtcattcctcaacccgctatc
tataagcctcgtcctcggtggactggtaagcagcttatcagcatggttatccctaaggag
gttagcctgttcaacggtacggattctggcgaaaacgcccctcttaaggacgagggtctt
ctgatccaagccggccaactgatgtatggtcttttaactaagaagaacattggtgctgct
gcgggtggtattgtgcatatcagctacaacgaacttggccccgaaggtgcgatggctttc
ttaaacggtgtccagcaggttgtcacctactggcttctcaacaatggtcatagcattggt
attggtgatacaattcccgatgcggcgaccattgctaaagttcaggtacatattgatgag
gaaaaggctgaagttgctcgcttgacagcaatggccacagcgaatgagcttgaggcccta
cctggtatgaacgttcgtgcaaccttcgaaaacaaagtctccatggctctgaaccaggcc
cgtgataaggctggtaccacaacacagaagagtttgaaggattcaaacaacgctgtcacc
atggcttcctcaggttccaagggttcatctatcaatatttctcaaatgactgcgcttgtc
ggtcagcaaattgtcgaaggcaagcgtattccttttggtttcaagtatcgcacattacct
cacttcaccaaggac
>GA_99.2 no comment
gagatgaaccttcacgtcccccagagtgaggaaacgcgcgctgaggttaaagagctttgt
ctagttcccttgaacattgtctctcctcagaagaacggtcctttgatgggtatcgtccag
gactctctggctggtgcctacaagctttgccgtcgagatgttttcctcacaaaggagcaa
atcatgaactgtatgctctgggtgcccaactgggacggtgtcattcctcaacccgctatc
tataagcctcgtcctcggtggactggtaagcagcttatcagcatggttatccctaaggag
gttagcctgttcaacggtacggattctggcgaaaacgcccctcttaaggacgagggtctt
ctgatccaagccggccaactgatgtatggtcttttaactaagaagaacattggtgctgct
gcgggtggtattgtgcatatcagctacaacgaacttggccccgaaggtgcgatggctttc
ttaaacggtgtccagcaggttgtcacctactggcttctcaacaatggtcatagcattggt
attggtgatacaattcccgatgcggcgaccattgctaaagttcaggtacatattgatgag
gaaaaggctgaagttgctcgcttgacagcaatggccacagcgaatgagcttgaggcccta
cctggtatgaacgttcgtgcaaccttcgaaaacaaagtctccatggctctgaaccaggcc
cgtgataaggctggtaccacaacacagaagagtttgaaggattcaaacaacgctgtcacc
atggcttcctcaggttccaagggttcatctatcaatatttctcaaatgactgcgcttgtc
ggtcagcaaattgtcgaaggcaagcgtattccttttggtttcaagtatcgcacattacct
cacttcaccaaggac
>NL_FOA01 no comment
gagatgaaccttcacgtcccccagagcgaggaaacgcgcgctgaggttaaagagctttgt
ctagttcccttgaacattgtctctcctcagaagaacggtcctttgatgggtatcgtccag
gactctctggctggtgcctacaagctttgccgtcgagatgttttcctcacaaaggagcaa
atcatgaactgtatgctctgggtgcccaactgggacggtgtcattcctcaacccgctatc
tataagcctcgtcctcggtggactggtaagcagcttatcagcatggttatccctaaggag
gttagcctgttcaacggtacggattctggtgaaaacgcccctcttaaggacgagggtctt
ctgatccaagccggccaactgatgtatggtcttttaactaagaagaacattggtgctgct
gcgggtggtattgtgcatatcagctacaacgaacttggccccgaaggtgcgatggctttc
ttaaacggtgtccagcaggttgtcacctactggcttctcaacaatggtcatagcattggt
attggtgatacaattcccgatgcggcgaccattgctaaagttcaggtacatattgatgag
gaaaaggctgaagttgctcgcttgacagcaatggccacagcgaatgagcttgaggcccta
cctggtatgaacgttcgtgcaaccttcgaaaacaaagtctccatggctctgaaccaggcc
cgtgataaggctggtaccacaacacagaagagtttgaaggattcaaacaacgctgtcacc
atggcttcctcaggttccaagggttcatctatcaatatttctcaaatgactgcgcttgtc
ggtcagcaaattgtcgaaggcaagcgtattccttttggtttcaagtatcgcacattacct
cacttcaccaaggac
>NL_FOA02 no comment
gagatgaaccttcacgtcccccagagcgaggaaacgcgcgctgaggttaaagagctttgt
ctagttcccttgaacattgtctctcctcagaagaacggtcctttgatgggtatcgtccag
gactctctggctggtgcctacaagctttgccgtcgagatgttttcctcacaaaggagcaa
atcatgaactgtatgctctgggtgcccaactgggacggtgtcattcctcaacccgctatc
tataagcctcgtcctcggtggactggtaagcagcttatcagcatggttatccctaaggag
gttagcctgttcaacggtacggattctggtgaaaacgcccctcttaaggacgagggtctt
ctgatccaagccggccaactgatgtatggtcttttaactaagaagaacattggtgctgct
gcgggtggtattgtgcatatcagctacaacgaacttggccccgaaggtgcgatggctttc
ttaaacggtgtccagcaggttgtcacctactggcttctcaacaatggtcatagcattggt
attggtgatacaattcccgatgcggcgaccattgctaaagttcaggtacatattgatgag
gaaaaggctgaagttgctcgcttgacagcaatggccacagcgaatgagcttgaggcccta
cctggtatgaacgttcgtgcaaccttcgaaaacaaagtctccatggctctgaaccaggcc

cgtgataaggctggtaccacaacacagaagagtttgaaggattcaaacaacgctgtcacc
atggcttcctcaggttccaagggttcatctatcaatatttctcaaatgactgcgcttgtc
ggtcagcaaattgtcgaaggcaagcgtattccttttggtttcaagtatcgcacattacct
cacttcaccaaggac
>NL_FOA03 no comment
gagatgaaccttcacgtcccccagagtgaggaaacgcgcgctgaggttaaagagctttgt
ctagttcccttgaacattgtctctcctcagaagaacggtcctttgatgggtatcgtccag
gactctctggctggtgcctacaagctttgccgtcgagatgttttcctcacaaaggagcaa
atcatgaactgtatgctctgggtgcccaactgggacggtgtcattcctcaacccgctatc
tataagcctcgtcctcggtggactggtaagcagcttatcagcatggttatccctaaggag
gttagcctgttcaacggtacggattctggcgaaaacgcccctcttaaggacgagggtctt
ctgatccaagccggccaactgatgtatggtcttttaactaagaagaacattggtgctgct
gcgggtggtattgtgcatatcagctacaacgaacttggccccgaaggtgcgatggctttc
ttaaacggtgtccagcaggttgtcacctactggcttctcaacaatggtcatagcattggt
attggtgatacaattcccgatgcggcgaccattgctaaagttcaggtacatattgatgag
gaaaaggctgaagttgctcgcttgacagcaatggccacagcgaatgagcttgaggcccta
cctggtatgaacgttcgtgcaaccttcgaaaacaaagtctccatggctctgaaccaggcc
cgtgataaggctggtaccacaacacagaagagtttgaaggattcaaacaacgctgtcacc
atggcttcctcaggttccaagggttcatctatcaatatttctcaaatgactgcgcttgtc
ggtcagcaaattgtcgaaggcaagcgtattccttttggtttcaagtatcgcacattacct
cacttcaccaaggac
>NL_FOA04 no comment
gagatgaaccttcacgtcccccagagtgaggaaacgcgcgctgaggttaaagagctttgt
ctagttcccttgaacattgtctctcctcagaagaacggtcctttgatgggtatcgtccag
gactctctggctggtgcctacaagctttgccgtcgagatgttttcctcacaaaggagcaa
atcatgaactgtatgctctgggtgcccaactgggacggtgtcattcctcaacccgctatc
tataagcctcgtcctcggtggactggtaagcagcttatcagcatggttatccctaaggag
gttagcctgttcaacggtacggattctggcgaaaacgcccctcttaaggacgagggtctt
ctgatccaagccggccaactgatgtatggtcttttaactaagaagaacattggtgctgct
gcgggtggtattgtgcatatcagctacaacgaacttggccccgaaggtgcgatggctttc
ttaaacggtgtccagcaggttgtcacctactggcttctcaacaatggtcatagcattggt
attggtgatacaattcccgatgcggcgaccattgctaaagttcaggtacatattgatgag
gaaaaggctgaagttgctcgcttgacagcaatggccacagcgaatgagcttgaggcccta
cctggtatgaacgttcgtgcaaccttcgaaaacaaagtctccatggctctgaaccaggcc
cgtgataaggctggtaccacaacacagaagagtttgaaggattcaaacaacgctgtcacc
atggcttcctcaggttccaagggttcatctatcaatatttctcaaatgactgcgcttgtc
ggtcagcaaattgtcgaaggcaagcgtattccttttggtttcaagtatcgcacattacct
cacttcaccaaggac
>NL_FOA05 no comment
gagatgaaccttcacgtcccccagagtgaggaaacgcgcgctgaggttaaagagctttgt
ctagttcccttgaacattgtctctcctcagaagaacggtcctttgatgggtatcgtccag
gactctctggctggtgcctacaagctttgccgtcgagatgttttcctcacaaaggagcaa
atcatgaactgtatgctctgggtgcccaactgggacggtgtcattcctcaacccgctatc
tataagcctcgtcctcggtggactggtaagcagcttatcagcatggttatccctaaggag
gttagcctgttcaacggtacggattctggcgaaaacgcccctcttaaggacgagggtctt
ctgatccaagccggccaactgatgtatggtcttttaactaagaagaacattggtgctgct
gcgggtggtattgtgcatatcagctacaacgaacttggccccgaaggtgcgatggctttc
ttaaacggtgtccagcaggttgtcacctactggcttctcaacaatggtcatagcattggt
attggtgatacaattcccgatgcggcgaccattgctaaagttcaggtacatattgatgag
gaaaaggctgaagttgctcgcttgacagcaatggccacagcgaatgagcttgaggcccta
cctggtatgaacgttcgtgcaaccttcgaaaacaaagtctccatggctctgaaccaggcc
cgtgataaggctggtaccacaacacagaagagtttgaaggattcaaacaacgctgtcacc
atggcttcctcaggttccaagggttcatctatcaatatttctcaaatgactgcgcttgtc
ggtcagcaaattgtcgaaggcaagcgtattccttttggtttcaagtatcgcacattacct
cacttcaccaaggac
>SP_FOA01 no comment
gagatgaaccttcacgtcccccagagtgaggaaacgcgcgctgaggttaaagagctttgt
ctagttcccttgaacattgtctctcctcagaagaacggtcctttgatgggtatcgtccag
gactctctggctggtgcctacaagctttgccgtcgagatgttttcctcacaaaggagcaa
atcatgaactgtatgctctgggtgcccaactgggacggtgtcattcctcaacccgctatc
tataagcctcgtcctcggtggactggtaagcagcttatcagcatggttatccctaaggag

gttagcctgttcaacggtacggattctggcgaaaacgcccctcttaaggacgagggtctt
ctgatccaagccggccaactgatgtatggtcttttaactaagaagaacattggtgctgct
gcgggtggtattgtgcatatcagctacaacgaacttggccccgaaggtgcgatggctttc
ttaaacggtgtccagcaggttgtcacctactggcttctcaacaatggtcatagcattggt
attggtgatacaattcccgatgcggcgaccattgctaaagttcaggtacatattgatgag
gaaaaggctgaagttgctcgcttgacagcaatggccacagcgaatgagcttgaggcccta
cctggtatgaacgttcgtgcaaccttcgaaaacaaagtctccatggctctgaaccaggcc
cgtgataaggctggtaccacaacacagaagagtttgaaggattcaaacaacgctgtcacc
atggcttcctcaggttccaagggttcatctatcaatatttctcaaatgactgcgcttgtc
ggtcagcaaattgtcgaaggcaagcgtattccttttggtttcaagtatcgcacattacct
cacttcaccaaggac
>SP_FOA02 no comment
gagatgaaccttcacgtcccccagagtgaggaaacgcgcgctgaggttaaagagctttgt
ctagttcccttgaacattgtctctcctcagaagaacggtcctttgatgggtatcgtccag
gactctctggctggtgcctacaagctttgccgtcgagatgttttcctcacaaaggagcaa
atcatgaactgtatgctctgggtgcccaactgggacggtgtcattcctcaacccgctatc
tataagcctcgtcctcggtggactggtaagcagcttatcagcatggttatccctaaggag
gttagcctgttcaacggtacggattctggcgaaaacgcccctcttaaggacgagggtctt
ctgatccaagccggccaactgatgtatggtcttttaactaagaagaacattggtgctgct
gcgggtggtattgtgcatatcagctacaacgaacttggccccgaaggtgcgatggctttc
ttaaacggtgtccagcaggttgtcacctactggcttctcaacaatggtcatagcattggt
attggtgatacaattcccgatgcggcgaccattgctaaagttcaggtacatattgatgag
gaaaaggctgaagttgctcgcttgacagcaatggccacagcgaatgagcttgaggcccta
cctggtatgaacgttcgtgcaaccttcgaaaacaaagtctccatggctctgaaccaggcc
cgtgataaggctggtaccacaacacagaagagtttgaaggattcaaacaacgctgtcacc
atggcttcctcaggttccaagggttcatctatcaatatttctcaaatgactgcgcttgtc
ggtcagcaaattgtcgaaggcaagcgtattccttttggtttcaagtatcgcacattacct
cacttcaccaaggac
>SP_FOA03 no comment
gagatgaaccttcacgtcccccagagtgaggaaacgcgcgctgaggttaaagagctttgt
ctagttcccttgaacattgtctctcctcagaagaacggtcctttgatgggtatcgtccag
gactctctggctggtgcctacaagctttgccgtcgagatgttttcctcacaaaggagcaa
atcatgaactgtatgctctgggtgcccaactgggacggtgtcattcctcaacccgctatc
tataagcctcgtcctcggtggactggtaagcagcttatcagcatggttatccctaaggag
gttagcctgttcaacggtacggattctggcgaaaacgcccctcttaaggacgagggtctt
ctgatccaagccggccaactgatgtatggtcttttaactaagaagaacattggtgctgct
gcgggtggtattgtgcatatcagctacaacgaacttggccccgaaggtgcgatggctttc
ttaaacggtgtccagcaggttgtcacctactggcttctcaacaatggtcatagcattggt
attggtgatacaattcccgatgcggcgaccattgctaaagttcaggtacatattgatgag
gaaaaggctgaagttgctcgcttgacagcaatggccacagcgaatgagcttgaggcccta
cctggtatgaacgttcgtgcaaccttcgaaaacaaagtctccatggctctgaaccaggcc
cgtgataaggctggtaccacaacacagaagagtttgaaggattcaaacaacgctgtcacc
atggcttcctcaggttccaagggttcatctatcaatatttctcaaatgactgcgcttgtc
ggtcagcaaattgtcgaaggcaagcgtattccttttggtttcaagtatcgcacattacct
cacttcaccaaggac
>SP_FOA04 no comment
gagatgaaccttcacgtcccccagagtgaggaaacgcgcgctgaggttaaagagctttgt
ctagttcccttgaacattgtctctcctcagaagaacggtcctttgatgggtatcgtccag
gactctctggctggtgcctacaagctttgccgtcgagatgttttcctcacaaaggagcaa
atcatgaactgtatgctctgggtgcccaactgggacggtgtcattcctcaacccgctatc
tataagcctcgtcctcggtggactggtaagcagcttatcagcatggttatccctaaggag
gttagcctgttcaacggtacggattctggcgaaaacgcccctcttaaggacgagggtctt
ctgatccaagccggccaactgatgtatggtcttttaactaagaagaacattggtgctgct
gcgggtggtattgtgcatatcagctacaacgaacttggccccgaaggtgcgatggctttc
ttaaacggtgtccagcaggttgtcacctactggcttctcaacaatggtcatagcattggt
attggtgatacaattcccgatgcggcgaccattgctaaagttcaggtacatattgatgag
gaaaaggctgaagttgctcgcttgacagcaatggccacagcgaatgagcttgaggcccta
cctggtatgaacgttcgtgcaaccttcgaaaacaaagtctccatggctctgaaccaggcc
cgtgataaggctggtaccacaacacagaagagtttgaaggattcaaacaacgctgtcacc
atggcttcctcaggttccaagggttcatctatcaatatttctcaaatgactgcgcttgtc
ggtcagcaaattgtcgaaggcaagcgtattccttttggtttcaagtatcgcacattacct

cacttcaccaaggac
>SP_FOA05 no comment
gagatgaaccttcacgtcccccagagtgaggaaacgcgcgctgaggttaaagagctttgt
ctagttcccttgaacattgtctctcctcagaagaacggtcctttgatgggtatcgtccag
gactctctggctggtgcctacaagctttgccgtcgagatgttttcctcacaaaggagcaa
atcatgaactgtatgctctgggtgcccaactgggacggtgtcattcctcaacccgctatc
tataagcctcgtcctcggtggactggtaagcagcttatcagcatggttatccctaaggag
gttagcctgttcaacggtacggattctggcgaaaacgcccctcttaaggacgagggtctt
ctgatccaagccggccaactgatgtatggtcttttaactaagaagaacattggtgctgct
gcgggtggtattgtgcatatcagctacaacgaacttggccccgaaggtgcgatggctttc
ttaaacggtgtccagcaggttgtcacctactggcttctcaacaatggtcatagcattggt
attggtgatacaattcccgatgcggcgaccattgctaaagttcaggtacatattgatgag
gaaaaggctgaagttgctcgcttgacagcaatggccacagcgaatgagcttgaggcccta
cctggtatgaacgttcgtgcaaccttcgaaaacaaagtctccatggctctgaaccaggcc
cgtgataaggctggtaccacaacacagaagagtttgaaggattcaaacaacgctgtcacc
atggcttcctcaggttccaagggttcatctatcaatatttctcaaatgactgcgcttgtc
ggtcagcaaattgtcgaaggcaagcgtattccttttggtttcaagtatcgcacattacct
cacttcaccaaggac
>SP_FOA06 no comment
gagatgaaccttcacgtcccccagagcgaggaaacgcgcgctgaggttaaagagctttgt
ctagttcccttgaacattgtctctcctcagaagaacggtcctttgatgggtatcgtccag
gactctctggctggtgcctacaagctttgccgtcgagatgttttcctcacaaaggagcaa
atcatgaactgtatgctctgggtgcccaactgggacggtgtcattcctcaacccgctatc
tataagcctcgtcctcggtggactggtaagcagcttatcagcatggttatccctaaggag
gttagcctgttcaacggtacggattctggtgaaaacgcccctcttaaggacgagggtctt
ctgatccaagccggccaactgatgtatggtcttttaactaagaagaacattggtgctgct
gcgggtggtattgtgcatatcagctacaacgaacttggccccgaaggtgcgatggctttc
ttaaacggtgtccagcaggttgtcacctactggcttctcaacaatggtcatagcattggt
attggtgatacaattcccgatgcggcgaccattgctaaagttcaggtacatattgatgag
gaaaaggctgaagttgctcgcttgacagcaatggccacagcgaatgagcttgaggcccta
cctggtatgaacgttcgtgcaaccttcgaaaacaaagtctccatggctctgaaccaggcc
cgtgataaggctggtaccacaacacagaagagtttgaaggattcaaacaacgctgtcacc
atggcttcctcaggttccaagggttcatctatcaatatttctcaaatgactgcgcttgtc
ggtcagcaaattgtcgaaggcaagcgtattccttttggtttcaagtatcgcacattacct
cacttcaccaaggac
>SP_FOA07 no comment
gagatgaaccttcacgtcccccagagtgaggaaacgcgcgctgaggttaaagagctttgt
ctagttcccttgaacattgtctctcctcagaagaacggtcctttgatgggtatcgtccag
gactctctggctggtgcctacaagctttgccgtcgagatgttttcctcacaaaggagcaa
atcatgaactgtatgctctgggtgcccaactgggacggtgtcattcctcaacccgctatc
tataagcctcgtcctcggtggactggtaagcagcttatcagcatggttatccctaaggag
gttagcctgttcaacggtacggattctggcgaaaacgcccctcttaaggacgagggtctt
ctgatccaagccggccaactgatgtatggtcttttaactaagaagaacattggtgctgct
gcgggtggtattgtgcatatcagctacaacgaacttggccccgaaggtgcgatggctttc
ttaaacggtgtccagcaggttgtcacctactggcttctcaacaatggtcatagcattggt
attggtgatacaattcccgatgcggcgaccattgctaaagttcaggtacatattgatgag
gaaaaggctgaagttgctcgcttgacagcaatggccacagcgaatgagcttgaggcccta
cctggtatgaacgttcgtgcaaccttcgaaaacaaagtctccatggctctgaaccaggcc
cgtgataaggctggtaccacaacacagaagagtttgaaggattcaaacaacgctgtcacc
atggcttcctcaggttccaagggttcatctatcaatatttctcaaatgactgcgcttgtc
ggtcagcaaattgtcgaaggcaagcgtattccttttggtttcaagtatcgcacattacct
cacttcaccaaggac
>SP_FOA08 no comment
gagatgaaccttcacgtcccccagagcgaggaaacgcgcgctgaggttaaagagctttgt
ctagttcccttgaacattgtctctcctcagaagaacggtcctttgatgggtatcgtccag
gactctctggctggtgcctacaagctttgccgtcgagatgttttcctcacaaaggagcaa
atcatgaactgtatgctctgggtgcccaactgggacggtgtcattcctcaacccgctatc
tataagcctcgtcctcggtggactggtaagcagcttatcagcatggttatccctaaggag
gttagcctgttcaacggtacggattctggtgaaaacgcccctcttaaggacgagggtctt
ctgatccaagccggccaactgatgtatggtcttttaactaagaagaacattggtgctgct
gcgggtggtattgtgcatatcagctacaacgaacttggccccgaaggtgcgatggctttc

ttaaacggtgtccagcaggttgtcacctactggcttctcaacaatggtcatagcattggt
attggtgatacaattcccgatgcggcgaccattgctaaagttcaggtacatattgatgag
gaaaaggctgaagttgctcgcttgacagcaatggccacagcgaatgagcttgaggcccta
cctggtatgaacgttcgtgcaaccttcgaaaacaaagtctccatggctctgaaccaggcc
cgtgataaggctggtaccacaacacagaagagtttgaaggattcaaacaacgctgtcacc
atggcttcctcaggttccaagggttcatctatcaatatttctcaaatgactgcgcttgtc
ggtcagcaaattgtcgaaggcaagcgtattccttttggtttcaagtatcgcacattacct
cactttaccaaggac
>SP_FOA09 no comment
gagatgaaccttcacgtcccccagagcgaggaaacgcgcgctgaggttaaagagctttgt
ctagttcccttgaacattgtctctcctcagaagaacggtcctttgatgggtatcgtccag
gactctctggctggtgcctacaagctttgccgtcgagatgttttcctcacaaaggagcaa
atcatgaactgtatgctctgggtgcccaactgggacggtgtcattcctcaacccgctatc
tataagcctcgtcctcggtggactggtaagcagcttatcagcatggttatccctaaggag
gttagcctgttcaacggtacggattctggtgaaaacgcccctcttaaggacgagggtctt
ctgatccaagccggccaactgatgtatggtcttttaactaagaagaacattggtgctgct
gcgggtggtattgtgcatatcagctacaacgaacttggccccgaaggtgcgatggctttc
ttaaacggtgtccagcaggttgtcacctactggcttctcaacaatggtcatagcattggt
attggtgatacaattcccgatgcggcgaccattgctaaagttcaggtacatattgatgag
gaaaaggctgaagttgctcgcttgacagcaatggccacagcgaatgagcttgaggcccta
cctggtatgaacgttcgtgcaaccttcgaaaacaaagtctccatggctctgaaccaggcc
cgtgataaggctggtaccacaacacagaagagtttgaaggattcaaacaacgctgtcacc
atggcttcctcaggttccaagggttcatctatcaatatttctcaaatgactgcgcttgtc
ggtcagcaaattgtcgaaggcaagcgtattccttttggtttcaagtatcgcacattacct
cactttaccaaggac
>SP_FOA10 no comment
gagatgaaccttcacgtcccccagagtgaggaaacgcgcgctgaggttaaagagctttgt
ctagttcccttgaacattgtctctcctcagaagaacggtcctttgatgggtatcgtccag
gactctctggctggtgcctacaagctttgccgtcgagatgttttcctcacaaaggagcaa
atcatgaactgtatgctctgggtgcccaactgggacggtgtcattcctcaacccgctatc
tataagcctcgtcctcggtggactggtaagcagcttatcagcatggttatccctaaggag
gttagcctgttcaacggtacggattctggtgaaaacgcccctcttaaggacgagggtctt
ctgatccaagccggccaactgatgtatggtcttttaactaagaagaacattggtgctgct
gcgggtggtattgtgcatatcagctacaacgaacttggccccgaaggtgcgatggctttc
ttaaacggtgtccagcaggttgtcacctactggcttctcaacaatggtcatagcattggt
attggtgatacaattcccgatgcggcgaccattgctaaagttcaggtacatattgatgag
gaaaaggctgaagttgctcgcttgacagcaatggccacagcgaatgagcttgaggcccta
cctggtatgaacgttcgtgcaaccttcgaaaacaaagtctccatggctctgaaccaggcc
cgtgataaggctggtaccacaacacagaagagtttgaaggattcaaacaacgctgtcacc
atggcttcctcaggttccaagggttcatctatcaatatttctcaaatgactgcgcttgtc
ggtcagcaaattgtcgaaggcaagcgtattccttttggtttcaagtatcgcacattacct
cacttcaccaaggac
>SP_FOA11 no comment
gagatgaaccttcacgtcccccagagcgaggaaacgcgcgctgaggttaaagagctttgt
ctagttcccttgaacattgtctctcctcagaagaacggtcctttgatgggtatcgtccag
gactctctggctggtgcctacaagctttgccgtcgagatgttttcctcacaaaggagcaa
atcatgaactgtatgctctgggtgcccaactgggacggtgtcattcctcaacccgctatc
tataagcctcgtcctcggtggactggtaagcagcttatcagcatggttatccctaaggag
gttagcctgttcaacggtacggattctggtgaaaacgcccctcttaaggacgagggtctt
ctgatccaagccggccaactgatgtatggtcttttaactaagaagaacattggtgctgct
gcgggtggtattgtgcatatcagctacaacgaacttggccccgaaggtgcgatggctttc
ttaaacggtgtccagcaggttgtcacctactggcttctcaacaatggtcatagcattggt
attggtgatacaattcccgatgcggcgaccattgctaaagttcaggtacatattgatgag
gaaaaggctgaagttgctcgcttgacagcaatggccacagcgaatgagcttgaggcccta
cctggtatgaacgttcgtgcaaccttcgaaaacaaagtctccatggctctgaaccaggcc
cgtgataaggctggtaccacaacacagaagagtttgaaggattcaaacaacgctgtcacc
atggcttcctcaggttccaagggttcatctatcaatatttctcaaatgactgcgcttgtc
ggtcagcaaattgtcgaaggcaagcgtattccttttggtttcaagtatcgcacattacct
cacttcaccaaggac
>SP_FOA12 no comment
gagatgaaccttcacgtcccccagagcgaggaaacgcgcgctgaggttaaagagctttgt

ctagttcccttgaacattgtctctcctcagaagaacggtcctttgatgggtatcgtccag
gactctctggctggtgcctacaagctttgccgtcgagatgttttcctcacaaaggagcaa
atcatgaactgtatgctctgggtgcccaactgggacggtgtcattcctcaacccgctatc
tataagcctcgtcctcggtggactggtaagcagcttatcagcatggttatccctaaggag
gttagcctgttcaacggtacggattctggtgaaaacgcccctcttaaggacgagggtctt
ctgatccaagccggccaactgatgtatggtcttttaactaagaagaacattggtgctgct
gcgggtggtattgtgcatatcagctacaacgaacttggccccgaaggtgcgatggctttc
ttaaacggtgtccagcaggttgtcacctactggcttctcaacaatggtcatagcattggt
attggtgatacaattcccgatgcggcgaccattgctaaagttcaggtacatattgatgag
gaaaaggctgaagttgctcgcttgacagcaatggccacagcgaatgagcttgaggcccta
cctggtatgaacgttcgtgcaaccttcgaaaacaaagtctccatggctctgaaccaggcc
cgtgataaggctggtaccacaacacagaagagtttgaaggattcaaacaacgctgtcacc
atggcttcctcaggttccaagggttcatctatcaatatttctcaaatgactgcgcttgtc
ggtcagcaaattgtcgaaggcaagcgtattccttttggtttcaagtatcgcacattacct
cacttcaccaaggac
>SP_FOA13 no comment
gagatgaaccttcacgtcccccagagtgaggaaacgcgcgctgaggttaaagagctttgt
ctagttcccttgaacattgtctctcctcagaagaacggtcctttgatgggtatcgtccag
gactctctggctggtgcctacaagctttgccgtcgagatgttttcctcacaaaggagcaa
atcatgaactgtatgctctgggtgcccaactgggacggtgtcattcctcaacccgctatc
tataagcctcgtcctcggtggactggtaagcagcttatcagcatggttatccctaaggag
gttagcctgttcaacggtacggattctggcgaaaacgcccctcttaaggacgagggtctt
ctgatccaagccggccaactgatgtatggtcttttaactaagaagaacattggtgctgct
gcgggtggtattgtgcatatcagctacaacgaacttggccccgaaggtgcgatggctttc
ttaaacggtgtccagcaggttgtcacctactggcttctcaacaatggtcatagcattggt
attggtgatacaattcccgatgcggcgaccattgctaaagttcaggtacatattgatgag
gaaaaggctgaagttgctcgcttgacagcaatggccacagcgaatgagcttgaggcccta
cctggtatgaacgttcgtgcaaccttcgaaaacaaagtctccatggctctgaaccaggcc
cgtgataaggctggtaccacaacacagaagagtttgaaggattcaaacaacgctgtcacc
atggcttcctcaggttccaagggttcatctatcaatatttctcaaatgactgcgcttgtc
ggtcagcaaattgtcgaaggcaagcgtattccttttggtttcaagtatcgcacattacct
cacttcaccaaggac
>SP_FOA14 no comment
gagatgaaccttcacgtcccccagagtgaggaaacgcgcgctgaggttaaagagctttgt
ctagttcccttgaacattgtctctcctcagaagaacggtcctttgatgggtatcgtccag
gactctctggctggtgcctacaagctttgccgtcgagatgttttcctcacaaaggagcaa
atcatgaactgtatgctctgggtgcccaactgggacggtgtcattcctcaacccgctatc
tataagcctcgtcctcggtggactggtaagcagcttatcagcatggttatccctaaggag
gttagcctgttcaacggtacggattctggcgaaaacgcccctcttaaggacgagggtctt
ctgatccaagccggccaactgatgtatggtcttttaactaagaagaacattggtgctgct
gcgggtggtattgtgcatatcagctacaacgaacttggccccgaaggtgcgatggctttc
ttaaacggtgtccagcaggttgtcacctactggcttctcaacaatggtcatagcattggt
attggtgatacaattcccgatgcggcgaccattgctaaagttcaggtacatattgatgag
gaaaaggctgaagttgctcgcttgacagcaatggccacagcgaatgagcttgaggcccta
cctggtatgaacgttcgtgcaaccttcgaaaacaaagtctccatggctctgaaccaggcc
cgtgataaggctggtaccacaacacagaagagtttgaaggattcaaacaacgctgtcacc
atggcttcctcaggttccaagggttcatctatcaatatttctcaaatgactgcgcttgtc
ggtcagcaaattgtcgaaggcaagcgtattccttttggtttcaagtatcgcacattacct
cacttcaccaaggac
>SP_FOA15 no comment
gagatgaaccttcacgtccctcagagtgaggaaacgcgcgctgaggttaaagagctttgt
ctagttcccttgaacattgtctctcctcagaagaacggtcctttgatgggtatcgtccag
gactctctggctggtgcctacaagctttgccgtcgagatgttttcctcacaaaggagcaa
atcatgaactgtatgctctgggtgcccaactgggacggtgtcattcctcaacccgctatc
tataagcctcgtcctcggtggactggtaagcagcttatcagcatggttatccctaaggag
gttagcctgttcaacggtacggattctggcgaaaacgcccctcttaaggacgagggtctt
ctgatccaagccggccaactgatgtatggtcttttaactaagaagaacattggtgctgct
gcgggtggtattgtgcatatcagctacaacgaacttggccccgaaggtgcgatggctttc
ttaaacggtgtccagcaggttgtcacctactggcttctcaacaatggtcatagcattggt
attggtgatacaattcccgatgcggcgaccattgctaaagttcaggtacatattgatgag
gaaaaggctgaagttgctcgcttgacagcaatggccacagcgaatgagcttgaggcccta

cctggtatgaacgttcgtgcaaccttcgaaaacaaagtctccatggctctgaaccaggcc
cgtgataaggctggtaccacaacacagaagagtttgaaggattcaaacaacgctgtcacc
atggcttcctcaggttccaagggttcatctatcaatatttctcaaatgactgcgcttgtc
ggtcagcaaattgttgaaggcaagcgtattccttttggtttcaagtatcgcacattacct
cacttcaccaaggac
>SP_FOA16 no comment
gagatgaaccttcacgtccctcagagtgaggaaacgcgcgctgaggttaaagagctttgt
ctagttcccttgaacattgtctctcctcagaagaacggtcctttgatgggtatcgtccag
gactctctggctggtgcctacaagctttgccgtcgagatgttttcctcacaaaggagcaa
atcatgaactgtatgctctgggtgcccaactgggacggtgtcattcctcaacccgctatc
tataagcctcgtcctcggtggactggtaagcagcttatcagcatggttatccctaaggag
gttagcctgttcaacggtacggattctggcgaaaacgcccctcttaaggacgagggtctt
ctgatccaagccggccaactgatgtatggtcttttaactaagaagaacattggtgctgct
gcgggtggtattgtgcatatcagctacaacgaacttggccccgaaggtgcgatggctttc
ttaaacggtgtccagcaggttgtcacctactggcttctcaacaatggtcatagcattggt
attggtgatacaattcccgatgcggcgaccattgctaaagttcaggtacatattgatgag
gaaaaggctgaagttgctcgcttgacagcaatggccacagcgaatgagcttgaggcccta
cctggtatgaacgttcgtgcaaccttcgaaaacaaagtctccatggctctgaaccaggcc
cgtgataaggctggtaccacaacacagaagagtttgaaggattcaaacaacgctgtcacc
atggcttcctcaggttccaagggttcatctatcaatatttctcaaatgactgcgcttgtc
ggtcagcaaattgttgaaggcaagcgtattccttttggtttcaagtatcgcacattacct
cacttcaccaaggac
>SP_FOA17 no comment
gagatgaaccttcacgtccctcagagtgaggaaacgcgcgctgaggttaaagagctttgt
ctagttcccttgaacattgtctctcctcagaagaacggtcctttgatgggtatcgtccag
gactctctggctggtgcctacaagctttgccgtcgagatgttttcctcacaaaggagcaa
atcatgaactgtatgctctgggtgcccaactgggacggtgtcattcctcaacccgctatc
tataagcctcgtcctcggtggactggtaagcagcttatcagcatggttatccctaaggag
gttagcctgttcaacggtacggattctggcgaaaacgcccctcttaaggacgagggtctt
ctgatccaagccggccaactgatgtatggtcttttaactaagaagaacattggtgctgct
gcgggtggtattgtgcatatcagctacaacgaacttggccccgaaggtgcgatggctttc
ttaaacggtgtccagcaggttgtcacctactggcttctcaacaatggtcatagcattggt
attggtgatacaattcccgatgcggcgaccattgctaaagttcaggtacatattgatgag
gaaaaggctgaagttgctcgcttgacagcaatggccacagcgaatgagcttgaggcccta
cctggtatgaacgttcgtgcaaccttcgaaaacaaagtctccatggctctgaaccaggcc
cgtgataaggctggtaccacaacacagaagagtttgaaggattcaaacaacgctgtcacc
atggcttcctcaggttccaagggttcatctatcaatatttctcaaatgactgcgcttgtc
ggtcagcaaattgttgaaggcaagcgtattccttttggtttcaagtatcgcacattacct
cacttcaccaaggac
>SP_FOA18 no comment
gagatgaaccttcacgtccctcagagtgaggaaacgcgcgctgaggttaaagagctttgt
ctagttcccttgaacattgtctctcctcagaagaacggtcctttgatgggtatcgtccag
gactctctggctggtgcctacaagctttgccgtcgagatgttttcctcacaaaggagcaa
atcatgaactgtatgctctgggtgcccaactgggacggtgtcattcctcaacccgctatc
tataagcctcgtcctcggtggactggtaagcagcttatcagcatggttatccctaaggag
gttagcctgttcaacggtacggattctggcgaaaacgcccctcttaaggacgagggtctt
ctgatccaagccggccaactgatgtatggtcttttaactaagaagaacattggtgctgct
gcgggtggtattgtgcatatcagctacaacgaacttggccccgaaggtgcgatggctttc
ttaaacggtgtccagcaggttgtcacctactggcttctcaacaatggtcatagcattggt
attggtgatacaattcccgatgcggcgaccattgctaaagttcaggtacatattgatgag
gaaaaggctgaagttgctcgcttgacagcaatggccacagcgaatgagcttgaggcccta
cctggtatgaacgttcgtgcaaccttcgaaaacaaagtctccatggctctgaaccaggcc
cgtgataaggctggtaccacaacacagaagagtttgaaggattcaaacaacgctgtcacc
atggcttcctcaggttccaagggttcatctatcaatatttctcaaatgactgcgcttgtc
ggtcagcaaattgttgaaggcaagcgtattccttttggtttcaagtatcgcacattacct
cacttcaccaaggac
>SP_FOA19 no comment
gagatgaaccttcacgtccctcagagtgaggaaacgcgcgctgaggttaaagagctttgt
ctagttcccttgaacattgtctctcctcagaagaacggtcctttgatgggtatcgtccag
gactctctggctggtgcctacaagctttgccgtcgagatgttttcctcacaaaggagcaa
atcatgaactgtatgctctgggtgcccaactgggacggtgtcattcctcaacccgctatc

```
tataagcctcgtcctcggtggactggtaagcagcttatcagcatggttatccctaaggag
gttagcctgttcaacggtacggattctggcgaaaacgcccctcttaaggacgagggtctt
ctgatccaagccggccaactgatgtatggtcttttaactaagaagaacattggtgctgct
gcgggtggtattgtgcatatcagctacaacgaacttggccccgaaggtgcgatggctttc
ttaaacggtgtccagcaggttgtcacctactggcttctcaacaatggtcatagcattggt
attggtgatacaattcccgatgcggcgaccattgctaaagttcaggtacatattgatgag
gaaaaggctgaagttgctcgcttgacagcaatggccacagcgaatgagcttgaggcccta
cctggtatgaacgttcgtgcaaccttcgaaaacaaagtctccatggctctgaaccaggcc
cgtgataaggctggtaccacaacacagaagagtttgaaggattcaaacaacgctgtcacc
atggcttcctcaggttccaagggttcatctatcaatatttctcaaatgactgcgcttgtc
ggtcagcaaattgttgaaggcaagcgtattccttttggtttcaagtatcgcacattacct
cacttcaccaaggac
>SP_FOA20 no comment
gagatgaaccttcacgtccctcagagtgaggaaacgcgcgctgaggttaaagagctttgt
ctagttcccttgaacattgtctctcctcagaagaacggtcctttgatgggtatcgtccag
gactctctggctggtgcctacaagctttgccgtcgagatgttttcctcacaaaggagcaa
atcatgaactgtatgctctgggtgcccaactgggacggtgtcattcctcaacccgctatc
tataagcctcgtcctcggtggactggtaagcagcttatcagcatggttatccctaaggag
gttagcctgttcaacggtacggattctggcgaaaacgcccctcttaaggacgagggtctt
ctgatccaagccggccaactgatgtatggtcttttaactaagaagaacattggtgctgct
gcgggtggtattgtgcatatcagctacaacgaacttggccccgaaggtgcgatggctttc
ttaaacggtgtccagcaggttgtcacctactggcttctcaacaatggtcatagcattggt
attggtgatacaattcccgatgcggcgaccattgctaaagttcaggtacatattgatgag
gaaaaggctgaagttgctcgcttgacagcaatggccacagcgaatgagcttgaggcccta
cctggtatgaacgttcgtgcaaccttcgaaaacaaagtctccatggctctgaaccaggcc
cgtgataaggctggtaccacaacacagaagagtttgaaggattcaaacaacgctgtcacc
atggcttcctcaggttccaagggttcatctatcaatatttctcaaatgactgcgcttgtc
ggtcagcaaattgttgaaggcaagcgtattccttttggtttcaagtatcgcacattacct
cacttcaccaaggac
```

S4: NEXUS partition file (annotation of exons/introns in TEF1A; RPB1,RPB2; coding only);

```
#nexus
begin sets;
    charset tef1_exons =  SP1_curated_partition_TEF1A.fasta: 185-323
379-408;
    charset tef1_intron1 = SP1_curated_partition_TEF1A.fasta: 1-184;
    charset tef1_intron2 = SP1_curated_partition_TEF1A.fasta: 324-378;
    charset rpb1 = SP2_curated_partition_RPB1.fasta: 1-915;
    charset rpb2 = SP3_curated_partition_RPB2.fasta: 1-853;
    charpartition mine = K2P+R2:tef1_intron1, K2P:tef1_intron2,
TNe+I:rpb1, TNe+I:rpb2;
end;
```

S5: Topology of the consensus tree obtained with IQ-TREE (Newick format).

```
(fusarium_proliferatum_et1_gca_900067095:0.1227398786,(((((((fusarium_
oxysporum_fo47_gca_000271705:0.0005105405,GASP_5:0.0039154154)37:0.000
5214953,(((((((((GASP_3:0.0000000000,GA_3.2:0.0000000000):0.0000000000
,GA_92.3:0.0000000000):0.0000000000,GA_5.4:0.0000000000):0.0000000000,
SP_FOA02:0.0000000000):0.0000000000,GA_4.2:0.0000000000):0.0000000000,
GA_5.3:0.0000000000):0.0000029556,GASP_9:0.0000029556)39:0.0000029556,
GA_99.2:0.0000029556)59:0.0000029556,GA_1.3:0.0000029556)63:0.00000208
22)24:0.0005062635,(GA_3.6:0.0005131070,(((((SP_FOA15:0.0000000000,SP_
FOA20:0.0000000000):0.0000000000,SP_FOA19:0.0000000000):0.0000000000,S
P_FOA18:0.0000000000):0.0000000000,SP_FOA17:0.0000000000):0.0000029556
,SP_FOA16:0.0000029556)97:0.0021059469)62:0.0005196686)31:0.0000024980
,(((((GASP_2:0.0000029556,GA_1.2:0.0000029556)38:0.0000029556,(GA_1.4
:0.0000029556,GA_3.5:0.0000029556)41:0.0000029556)74:0.0005079791,((GA
```

_10:0.0000029556,(((((GA_3.3:0.0000000000,SP_FOA01:0.0000000000):0.000
0000000,SP_FOA05:0.0000000000):0.0000000000,SP_FOA03:0.0000000000):0.0
000000000,SP_FOA04:0.0000000000):0.0000029556,GA_3.7:0.0000029556)69:0
.0000029556)77:0.0000029556,(GA_4.1:0.0000029556,GA_4.3:0.0000029556)9
7:0.0005085707)90:0.0000029556)85:0.0010269858,SP_FOA14:0.0000029556)3
6:0.0005067835,SP_FOA13:0.0005075787)19:0.0000029556,(NL_FOA03:0.00000
29556,NL_FOA04:0.0000029556)56:0.0000029556)13:0.0000029556)65:0.00050
67744,SP_FOA10:0.0000029556)67:0.0005067135,SP_FOA06:0.0000029556)82:0
.0006031118,GASP_4:0.0022766468)86:0.0005408163,((((((NL_FOA01:0.00000
29556,NL_FOA02:0.0000029556)85:0.0000020234,(NL_FOA05:0.0000020365,SP_
FOA07:0.0021770363)66:0.0010297728)78:0.0005286509,SP_FOA11:0.00000295
56)57:0.0000029556,SP_FOA12:0.0005068463)77:0.0005073258,SP_FOA08:0.00
00029556)55:0.0005073082,SP_FOA09:0.0000029556)84:0.0026767472);
